# Why Parents Say No to Having Their Children Vaccinated against Measles: A Systematic Review of the Social Determinants of Parental Perceptions on MMR Vaccine Hesitancy

**DOI:** 10.3390/vaccines11050926

**Published:** 2023-05-02

**Authors:** M. Lelinneth B. Novilla, Michael C. Goates, Alisha H. Redelfs, Mallory Quenzer, Lynneth Kirsten B. Novilla, Tyler Leffler, Christian A. Holt, Russell B. Doria, Michael T. Dang, Melissa Hewitt, Emma Lind, Elizabeth Prickett, Katelyn Aldridge

**Affiliations:** 1 Department of Public Health, Brigham Young University, Provo, UT 84602, USA; 2 Harold B. Lee Library, Brigham Young University, Provo, UT 84602, USA; 3 School of Osteopathic Medicine, Campbell University, Lillington, NC 27546, USA

**Keywords:** measles, MMR vaccine, measles vaccine, vaccine hesitancy, social determinants of health

## Abstract

Ongoing outbreaks of measles threaten its elimination status in the United States. Its resurgence points to lower parental vaccine confidence and local pockets of unvaccinated and undervaccinated individuals. The geographic clustering of hesitancy to MMR indicates the presence of social drivers that shape parental perceptions and decisions on immunization. Through a qualitative systematic review of published literature (*n* = 115 articles; 7 databases), we determined major themes regarding parental reasons for MMR vaccine hesitancy, social context of MMR vaccine hesitancy, and trustworthy vaccine information sources. Fear of autism was the most cited reason for MMR hesitancy. The social drivers of vaccine hesitancy included primary care/healthcare, education, economy, and government/policy factors. Social factors, such as income and education, exerted a bidirectional influence, which facilitated or hindered vaccine compliance depending on how the social determinant was experienced. Fear of autism was the most cited reason for MMR hesitancy. Vaccine hesitancy to MMR and other childhood vaccines clustered in middle- to high-income areas among mothers with a college-level education or higher who preferred internet/social media narratives over physician-based vaccine information. They had low parental trust, low perceived disease susceptibility, and were skeptical of vaccine safety and benefits. Combating MMR vaccine misinformation and hesitancy requires intersectoral and multifaceted approaches at various socioecological levels to address the social drivers of vaccine behavior.

## 1. Introduction

Vaccines are the most effective public health measures against infectious diseases. Measles, a highly contagious acute viral respiratory infection, is a major cause of mortality and morbidity, particularly among children younger than five [1,2]. It can lead to serious complications such as pneumonia, encephalitis, diarrhea, dehydration, ear infections, and irreversible vision loss [1,3]. These complications are common among infants and young children who are malnourished or have compromised immune systems [3]. Measles is primarily transmitted through large respiratory droplets via coughing and/or sneezing or through aerosolized particles that remain airborne for up to 2 h in enclosed spaces [4,5]. Within seven to fourteen days from the time of exposure, a susceptible individual develops fever accompanied by the classic three “Cs” of measles: cough, coryza, and conjunctivitis [4,5]. Because of continued viral shedding from the infected individual, measles can be transmitted four days before and four days from the appearance of a maculopapular rash [4,5]. Given its high transmissibility, more than 90% of susceptible individuals around an infected person will develop measles [4,5]. 

The measles, mumps, and rubella (MMR) vaccine protects individuals, families, and communities. Prior to the introduction of MMR, the United States experienced almost four million cases of measles annually, resulting in 400 to 500 deaths, 48,000 hospitalizations, and 1000 patients with encephalitis [6]. In 1971, the US government approved Merck’s MMR combination vaccine that reduced the need for individual injections. Its adverse effects were no more than those seen in single vaccines and provided 96%, 95%, and 94% protection against measles, mumps, and rubella, respectively [7]. With the widespread rollout of MMR, measles cases in the US plummeted by more than 99%, resulting in its subsequent elimination in 2000 [7]. To maintain herd immunity, the Centers for Disease Control and Prevention (CDC) recommends a 95% or higher immunization coverage with a two-dose MMR series at 12–15 months and at 4–6 years of age [6,8].

MMR has been proven to be safe and effective, yet outbreaks continue to occur in multiple parts of the world. Gaps in vaccination, attributed to parental anxiety regarding autism that was erroneously linked to MMR, generated fear and promoted vaccine delays and refusals, thus, increasing the number of susceptible individuals [3,9,10,11,12,13]. From 2000 to 2017, the global coverage rates of the measles-containing-vaccine first dose (MCV1) and second dose (MCV2) stalled at 85% and 67%, respectively, instead of the 95% target needed to prevent outbreaks [3,13,14]. In addition, complacency toward measles transmission, and vaccine misinformation on top of an overburdened healthcare system, resulted in severe and prolonged measles outbreaks in multiple countries [3]. These led to a 31% global increase in reported cases of measles in 2016–2017 that were particularly high in three World Health Organization’s (WHO) regions: Americas (6358%), Eastern Mediterranean Region (481%), and Europe (458%), except in the Western Pacific Region where cases fell by 82% [3,13,14]. 

The increasing trend in measles cases globally persisted during the COVID-19 pandemic, with provisional data showing measles surges in several parts of the world in 2021 and 2022 [13,15]. Measles immunization continued to decline due to pandemic-related vaccine shortages and distribution issues that disrupted routine immunization programs in various parts of the world [15]. Additional factors included poor measles surveillance and persistent measles outbreaks. In 2021, nine million cases of measles and 128,000 measles-related deaths were reported globally [15]. Based on a joint CDC and WHO report, in 2021, 25 million children missed the first measles vaccine dose while another 14.7 million children missed the second dose, rendering many unprotected against measles [15]. Considering the regional variations in vaccination coverage, this presents a serious threat given the significantly high transmission potential of measles wherever there are pockets of unvaccinated or undervaccinated individuals—locally, nationally, and internationally. Due to gaps in vaccination, none of the WHO regions could sustain measles elimination with the re-introduction of measles in the population due to gaps in vaccination. This is concerning as foreign measles cases have historically entered the US through travel [13]. For instance, the 2019 measles outbreaks in the US were all linked to travel-related cases, which in turn, infected pockets of undervaccinated and/or unvaccinated communities [14].

### 1.1. Vaccine Hesitancy 

The World Health Organization’s (WHO) Strategic Advisory Group of Experts on Immunization (SAGE Working Group) defines vaccine hesitancy as the “delay in acceptance or refusal of vaccination despite availability of vaccination services” [9,10,11,12,16]. Although commonly exhibited as doubting and/or questioning of certain vaccines, vaccine hesitancy manifests in many forms: delaying early immunizations, choosing to limit shots per visit, avoiding certain vaccines, and failing to catch up on missed immunizations [9,10,11,12,16].

Trust and risk perception issues underlie the hesitancy to MMR and other childhood vaccines [17,18]. Trust is influenced by the interactions between “external levers of trust” (generalized trust, historical trust, influences outside the healthcare system) and vaccine-related factors (trust in product, provider, and policymaker) [19]. The greater the alignment between these factors, the higher the perceived level of trust. However, when trust erodes—on the vaccine, healthcare provider, or policymaker, then external influences would have a greater sway on vaccine decisions such as family, friends, neighbors, religious and political leaders, celebrities, including historical accounts of abuse and neglect by the government, pharmaceutical companies, or the healthcare system. These influences may drive the dynamics toward vaccine mistrust and away from expert opinion and scientific evidence, eventually dissuading parents from getting their children vaccinated [17,18,19]. On the other hand, the perception of risk is dependent upon the trust ascribed to the messenger and the message, the language and images used, and the level of fear incited by the message [17,20]. Professional and informal sources of vaccine information influence how parents perceive the safety and benefits of vaccines while language and images used in vaccine messages can incite positive or negative sentiments [20]. Fear, generated by vaccine misinformation, can erroneously amplify the erroneous perception of risk and can cast doubts on the science behind vaccine development. This is exemplified by parental concerns over vaccine contents, such as thimerosal, instead on the diseases that vaccines prevent [11,16]. There is the mistaken belief that being infected is more beneficial than having their children experience adverse vaccine effects, which are far more infrequent than catching the disease and experiencing its complications [20]. Thus, science-based messaging, particularly if communicated by messengers trusted by parents, such as physicians, has the potential to promote an accurate perception of the safety and efficacy of MMR and other childhood vaccines.

### 1.2. Social Determinants of MMR Vaccination

MMR vaccine hesitancy has sociocultural and geographical clustering elements. Local patterns of vaccine hesitancy is demonstrated by community clusters of nonmedical exemptions (NMEs), particularly against MMR [21]. Where NMEs for philosophical and religious reasons are high, MMR vaccine uptake is lower [17,21,22,23]. During the 2018–2019 school year, only 20 states reported a 95% or greater MMR vaccine coverage, while Idaho and Colorado remained below 90% [24]. In contrast, states with stringent NME policies for school entry had higher MMR vaccine uptake [17,21,22,23]. This spatial distribution of vaccine hesitancy follows societal structures and social, economic, educational, and political conditions, which are collectively referred to as the social determinants of health (SDH). The resulting socioeconomic and political milieu influence the distribution of “power, money, and resources” and can lead to systemic, unfair, and avoidable inequities in health status across population groups [18,25].

The social determinants of health are subdivided into intermediary and structural elements. Intermediary determinants include daily needs, early childhood circumstances, and the availability of and access to assets and resources. In turn, these intermediary determinants are shaped by socioeconomic status, employment, income, education, healthcare insurance, race/ethnicity, religion, and political ideologies/affiliation [18,25]. On the other hand, structural determinants include societal-level factors such as governance, policies, politics, geopolitical units, values, cultural norms, and biases.

One’s social environment, comprising the nested contexts of socioecological influences, bidirectionally shape individual vaccine beliefs, attitudes, and behavior just as these can influence societal norms [26]. Various cognitive, emotional, and contextual elements interact at multiple socioecological levels to influence vaccine beliefs and behavior [26,27]. At the individual level, one’s social network can persuade, encourage, and reinforce vaccination decisions but it can also dissuade from complying with recommended vaccine schedules. Thus, social drivers can either increase or diminish parental-level MMR vaccine confidence, compliance, and institutional trust [17]. For example, in deconstructing the 2015 Disneyland and 2019 New York measles outbreaks, root causes pointed to both parental perceptions and social factors that promoted vaccine delays, alternative schedules, refusals, and NMEs [17,21]. The reciprocal determinism illustrated by the bidirectional interactions between parental perceptions and societal factors affirms the context-specific nature of vaccine hesitancy [21].

Understanding the social determinants of MMR vaccine hesitancy can help inform approaches to vaccine messaging and vaccine compliance. However, gaps still remain in understanding and addressing the SDH context of MMR vaccine hesitancy. Through a qualitative analysis of the published literature, the present systematic review contributes to advancing the field by determining major themes regarding the following research questions:

1. Why do parents delay, refuse, or oppose to having their children vaccinated against measles and other vaccine preventable diseases?
2. What are the parental perceptions, attitudes, and practices on measles vaccination?
3. Where do parents/caregivers obtain measles vaccine information? Which source do parents/caregivers trust on measles immunization?
4. Which social factors impact parental perceptions, attitudes, and practices promote or hinder measles vaccine hesitancy?
5. How can families, primary care, health care, public health, and government address measles vaccine hesitancy?

This comprehensive review highlights recent developments, but more importantly, it specifically considers the social contributors of MMR hesitancy. In this paper, we discuss the implications for policy and practice and call for intersectoral collaborations across multiple socioecological levels to address hesitancy to MMR and to other childhood vaccines.

## 2. Materials and Methods

### 2.1. Search Strategy

The methodology for this qualitative systematic review followed the Preferred Reporting Items for Systematic Reviews and Meta-Analyses (PRISMA) guidelines [28]. Supplementary Materials Table S1 provides the search terms and databases searched in this systematic review.

### 2.2. Eligibility Criteria

Articles were included in this review if they met the following inclusion criteria:

- Written in English;
- Published during the years from 2000 to 2022 to capture patterns in vaccine hesitancy and vaccine perspectives, attitudes, and behavior within the context of the social determinants of health to help explain the resurgence in measles during the post-measles elimination era in the United States;
- Involved empirical research studies and/or literature reviews (journal articles, dissertations, theses);
- Focused on the United States;
- Addressed measles/MMR vaccine hesitancy;
- Discussed/mentioned parent/caregiver demographics, vaccine beliefs, attitudes, practices toward measles/MMR, sources of vaccine information, and social factors facilitating or hindering vaccine compliance.

For the purpose of this systematic review, we focused primarily on MMR. In our evaluation of MMR and childhood vaccine hesitancy, COVID-19 vaccines were not included because they were not approved by the FDA for administration in children until 17 June 2022. As such, the time frame in which articles on COVID-19 vaccine hesitancy in children would be published are outside the scope of this review.

### 2.3. Data Extraction and Management 

The same inclusion criteria were applied in the full-text evaluation to determine article suitability for qualitative analysis. Strategies, approaches, and recommendations for vaccine hesitancy from the articles included in the review were classified into primary care/healthcare, public health, and government levels based on Hillemeier et al.’s [29] directory of the 12 dimensions of social determinants, which defined each social determinant based on existing datasets that are used for quantifying each dimension at the local levels. Below are brief explanations of the social determinants used in coding for the qualitative analysis:

- Economy (annual household income and community poverty levels);
- Employment (job status, work–life balance);
- Education (attainment of formal education, access to health education materials);
- Political (legislation, political involvement);
- Environmental (neighborhood physical characteristics, environmental influences);
- Housing (home ownership, housing condition);
- Medical (physical distance to healthcare facilities, positive relationship with healthcare providers, access to health insurance);
- Governmental (funding, policy/legislation, services, local governments, civic participation, political structure, community organizations);
- Public health (health policy, intervention strategies);
- Psychosocial (influence of friends, extended family, other social networks);
- Behavioral (smoking, physical activity, diet/obesity, fresh fruit and vegetable consumption, alcohol/illicit drug use, violence);
- Transport (public transportation, personal vehicle ownership).

A detailed codebook (see Supplementary Material, Figure S1) was developed in advance to guide coders in extracting data and in identifying themes for the qualitative analysis. 

The full-text review was conducted by ten individual coders. Prior to the full-text review, all ten coders completed several rounds of test coding. Each test round used a sample of 10 or 15 articles that were independently evaluated by all coders to assess the accuracy and usability of the codebook and to determine the level of agreement among the coders. After each test round, each coder’s evaluation was compared to those of the other coders. Discrepancies in coding were discussed in team meetings to calibrate coders to the codebook and to reach consensus on coding results. Minor edits, as discussed during team meetings, were made to the codebook based on user experience. A total of 35 articles were evaluated during the initial rounds of test coding, after which, the remaining articles were divided among ten individual coders for a full-text review. Each article was randomly assigned to three independent coders who recorded their results using Qualtrics (Qualtrics, Provo, UT, USA). Once an article had been reviewed separately by three independent coders, the results for that article were discussed by the entire team during weekly research meetings. When discrepancies were noted, the entire team evaluated that article until a consensus was reached on the appropriate results. 

### 2.4. Data Analysis 

Following the abstract and full-text reviews based on the inclusion criteria, a thematic analysis was conducted to determine the major themes regarding MMR vaccine hesitancy. In addition to analyzing reasons for vaccine hesitancy, this thematic analysis also evaluated parental characteristics associated with vaccine hesitancy, vaccine information sources used by parents, as well as approaches to address vaccine hesitancy. Each of the included articles was evaluated and the results were presented in a tabular format. The quality of included articles was assessed using the Mixed Methods Appraisal Tool (MMAT) version 2018 [30]. Results of the MMAT quality analysis are available in the Supplementary Materials Table S2.

## 3. Results

**Figure 1:**
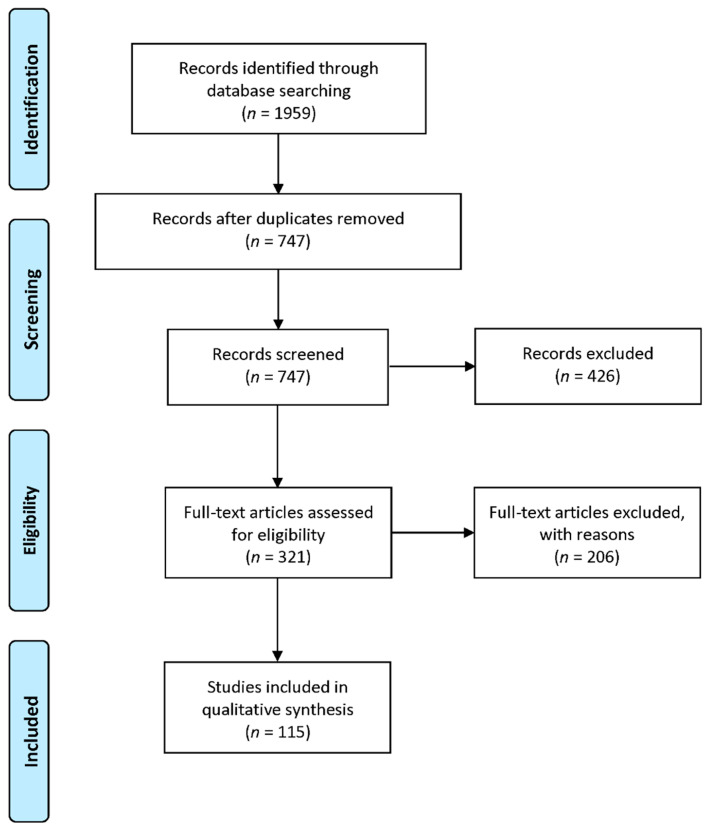
PRISMA diagram of the articles analyzed in the systematic review.

The final search yielded 1959 articles, which were reduced to 747 articles after removing duplicate articles. Using the inclusion criteria, the lead author conducted a title/abstract screening of all 747 unique articles, of which 321 articles were eligible for full-text review. After the full-text review, a total of 115 articles met the inclusion criteria and were included in the qualitative synthesis (see Figure 1 for the PRISMA diagram). For simplicity in reporting the results, the term “parents” includes parents, guardians, or caregivers.

### 3.1. Study Design and Vaccine Focus

Articles published from 2000 to 2022 on measles/MMR vaccine hesitancy that met the inclusion criteria were primarily surveys and literature reviews. Of the 115 articles included in this thematic analysis, 56 (49%) had quantitative research designs, 42 (37%) were literature reviews/conceptual papers, 10 (9%) were qualitative analyses, and seven (6%) used mixed-methods analyses. In terms of vaccine focus, the search terms yielded a mix of articles that were solely focused on measles/MMR hesitancy (13 of 115; 11%) while a majority mentioned hesitancy to both measles/MMR and other childhood vaccines (102 of 115; 89%). The large majority of studies that investigated MMR vaccine hesitancy were in concert with the analysis of other childhood vaccines. Supplementary Materials Table S3 provides additional information on the characteristics of the included studies [11,12,22,23,31,32,33,34,35,36,37,38,39,40,41,42,43,44,45,46,47,48,49,50,51,52,53,54,55,56,57,58,59,60,61,62,63,64,65,66,67,68,69,70,71,72,73,74,75,76,77,78,79,80,81,82,83,84,85,86,87,88,89,90,91,92,93,94,95,96,97,98,99,100,101,102,103,104,105,106,107,108,109,110,111,112,113,114,115,116,117,118,119,120,121,122,123,124,125,126,127,128,129,130,131,132,133,134,135,136,137,138,139,140,141].

### 3.2. Sociodemographics

Of the 115 articles included in this thematic analysis, 28 (24%) reported information on parental age. Seven of the 28 articles correlated older parental age with greater vaccine hesitancy or higher childhood vaccine exemption rates. Four articles associated younger parental age with greater vaccine hesitancy. Forty-two articles (37%) reported information on parental race or ethnicity, 15 of which found non-Hispanic White parents as more likely to be vaccine hesitant or to have undervaccinated children while 11 articles identified non-White parents as more likely to have undervaccinated children. 

Of the 115 articles, 34 (30%) mentioned maternal education as a control, a dependent variable, or as a finding of previous studies on vaccine hesitancy. Seventeen of the 34 articles (50%) mentioned the association between higher maternal education (i.e., some college or higher) and vaccine hesitancy, under- or unvaccination, and/or NMEs. In contrast, 15 of the 34 articles (44%) cited the association of lower parental education (high school or less than 12 years of formal education) with vaccine hesitancy, under- or unvaccination, and/or NMEs. 

In terms of income, 32 of the 115 articles (28%) mentioned income within the context of a dependent variable, a control, or as a finding of previous studies on socioeconomic status and vaccine hesitancy. Of the 32 articles, 17 (53%) specifically mentioned the association between higher annual household income (equal to or greater than $50,000/year) and vaccine hesitancy, under- or unvaccination, and/or NMEs. On the other hand, 11 of the 32 articles (34%) mentioned the association between lower annual household income with vaccine hesitancy, and under- or unvaccination. Only 11 of the 115 articles (10%) reported health insurance status. One article mentioned the association between health insurance coverage with higher vaccination rates. Another article identified higher vaccine hesitancy among those with private health insurance when compared to those with public insurance or no insurance. Conversely, another study found lower vaccination rates among individuals with public insurance or no insurance. One article associated lack of insurance with undervaccination patterns. 

Of the 115 articles, nine (8%) linked political affiliation/ideology to vaccine hesitancy, of which one article found that conservative respondents had higher levels of mistrust in health institutions and were less likely to vaccinate against measles and other vaccine-preventable diseases. Of the 115 articles, 16 (14%) connected marital status to vaccine hesitancy, six of which (38%) reported that married parents were more likely to be vaccine-hesitant or noncompliant while two (13%) found that children of single mothers were more likely to be undervaccinated. See Table 1 for the summary on vaccine hesitancy and parental demographic variables. For additional details on included studies on vaccine hesitancy and parental demographic variables, see Supplementary Materials Table S4.

### 3.3. Vaccine Hesitancy Themes

Authors of the articles included in this review mentioned the hesitancy to the MMR vaccine along with the hesitancy to other childhood vaccines. For example, 86 of the 115 articles (75%) identified risk of adverse/hypersensitivity reactions as the most common parental concern to childhood vaccines in general, followed by general/other safety concerns (54 of 115 articles; 47%), risk of autism (49 of 115 articles; 43%), and too many vaccines per clinic visit or concerns toward vaccine schedules (49 of 115 articles; 43%; see Table 2).

### 3.4. Vaccine Information Sources

Parents used several sources to obtain vaccine information. Primary care/healthcare, such as doctors and/or school nurses, was the most common source of vaccine information regardless of whether parents were vaccine compliant or vaccine hesitant. However, vaccine-hesitant parents (52 of 115 articles; 45%) and parents whose vaccine views were not specified by the authors (52 of 115 articles; 45%) still used the internet and social media more frequently compared to vaccine-compliant parents (25 of 115 articles; 22%). 

In terms of reliability of vaccine information, vaccine-compliant parents, vaccine-hesitant parents, and parents with nonspecified vaccine views ranked primary/healthcare sources as the most reliable source of information. Vaccine-compliant (46 of 115 articles; 40%) and parents with nonspecified views (45 of 115 articles; 39%) rated physician-based vaccine sources higher on trustworthiness as opposed to vaccine-hesitant parents (31 of 115 articles; 27%; see Table 3 and Table 4).

### 3.5. SDH Facilitating and Hindering Vaccine Compliance

The most cited social determinants facilitating vaccine compliance were primary care/healthcare (46 of 115 articles; 40%), education (30 of 115 articles; 26%), government/political (21 of 115 articles; 18%), and psychosocial/behavioral (20 of 115 articles; 17%). Other social factors mentioned included public health, economy/income, environmental/built environment, housing, employment, and transportation.

The most frequently cited social determinants that hindered vaccine compliance were psychosocial/behavioral (47 of 115 articles; 41%), primary care/healthcare (38 of 115 articles; 33%), and education (38 of 115 articles; 33%; see Table 5). 

### 3.6. Approaches to Vaccine Hesitancy

Studies included in this review either recommended or cited various approaches to vaccine hesitancy, which we classified as to primary care/healthcare, public health, and government-level strategies.

#### 3.6.1. Primary Care/Healthcare-Level Strategies

The majority of the articles (79 of 115 articles; 69%) mentioned approaches at the primary care/healthcare level such as conducting educational interventions in hospital settings (32 articles), improving vaccine communication methods in healthcare settings (22 articles), creating positive and trusting parent–physician relationships (16 articles), partnering with community entities (8 articles), improving vaccine-tracking records (8 articles), training physicians and clinical staff on the latest vaccine safety information (7 articles), and providing financial incentives or removing barriers to vaccination (4 articles). Of the 115 articles, 16 (14%) mentioned primary care/healthcare strategies specific to measles/MMR vaccine hesitancy such as communication training (8 articles), creating positive and trusting parent–physician relationships (7 articles), increasing patient–physician interaction time (4 articles), and training physicians and clinical staff on the latest vaccine safety information (1 article).

The present review originally classified approaches to vaccine hesitancy into either primary care or healthcare as to provider specialty and settings of practice based on Hillemeier et al.’s definition of primary care as to the number of providers, training/competence/certification, Medicaid/Medicare reimbursement levels [29]. In addition, considering the interactions of parents with school nurses, nurse practitioners, primary care physicians, pediatricians, or family physicians in an outpatient clinic versus a hospital or tertiary care setting, we also used the WHO’s definition of primary care to guide the systematic review, as the “first level of contact of individuals, the family, and community with the national health system” and the “first elements of a continuing health care process” [142]. However, we eventually merged the “primary care” and “healthcare” classifications because the articles included in this review broadly referred to “doctors,” “physicians,” or “parent–physician” relationships without always specifying the practice setting and/or provider specialty to sufficiently distinguish between the two categories. Further, several approaches cited were applicable in both primary and tertiary care settings.

#### 3.6.2. Public-Health-Level Strategies

Of the one hundred and fifteen articles, seventy-five (65%) discussed public health approaches to general vaccine hesitancy while 21 (18%) were specific to measles/MMR vaccine hesitancy. Additionally, 43 of the 115 articles (37%) discussed educational interventions, 27 (23%) focused on communication strategies, 18 (16%) emphasized approaches to specific concerns or audiences, 12 (10%) recommended a mix of traditional sources and social media for disseminating vaccine information, and nine (8%) mentioned improving access to vaccines.

#### 3.6.3. Government-Level Strategies

There were 48 articles out of 115 (42%) that identified government-level strategies toward general vaccine hesitancy while 12 (10%) were specific to measles/MMR vaccine hesitancy. These strategies included legislative or policy actions on eliminating religious/philosophical exemptions, creating stricter immunizations laws, or levying taxes on NMEs (32 of 115 articles; 28%). Six articles (5%) discussed the lack of trust in government sources and suggested partnering with trusted personalities or community icons.

## 4. Discussion

### 4.1. Themes on MMR Vaccine Hesitancy

Parental concerns regarding MMR continued to be influenced by the fear of autism (see Table 2). This concern appeared repeatedly throughout the literature regardless of the type of study—suggesting how powerful and lasting false information can persist in parents’ minds despite MMR’s established safety and efficacy. Additional reasons for parental hesitancy to MMR overlapped with the reasons for why parents also delayed or refused other childhood vaccines that it was not always easy to categorize vaccine hesitancy to MMR versus other vaccines.

The concern for autism gained particular traction among ethnic groups. For instance, Bahta and Ashkir [85] highlighted the erroneous perception of the link between MMR and autism in the Somali community. Given the disproportionately high cases of Autism Spectrum Disorder among Somalis in Hennepin County, Minnesota, fear of autism was pervasive even among highly educated Somalis who expressed their resistance to the MMR vaccine by saying: “My children did not get the MMR; my evidence is the Somali children I see who have autism,” or “I vaccinate except for the triple-letter vaccine” [85]. Similar views were noted among immigrant communities in Washington State. Foreign-born mothers from Ukraine, Russia, Somalia, and Mexico were more likely to be averse to the MMR vaccine, have underimmunized children, and attend fewer prenatal visits compared to parents who were born in the US [83].

Philosophical, moral, and/or religious objections played a role in vaccine hesitancy toward the MMR vaccine and/or other childhood vaccines (see Table 2). The correlation between high NMEs for philosophical, moral, or religious reasons and low immunization rates was repeatedly emphasized in several studies [11,135,136]. States with strict NME policies had fewer exemptions and a higher vaccine coverage compared to those with less stringent requirements [11,23,37,129,135]. 

Measles outbreaks were not randomly distributed. The ideological and spatial clustering of NMEs in measles hotspots indicated the impact of social processes that promoted parental hesitancy to the MMR vaccine and/or other childhood vaccines [22,23,37,60,129]. To illustrate, vaccine-hesitant viewpoints, that encouraged NMEs, appealed to certain demographics in association with ethnicity, income, education, or healthcare access [12,22,23,37,58,61,78,80,122,129,134]. Previous measles outbreaks congregated in kindergarten schools in California with lenient NME policies [21,22,23]. Thus, NMEs served as the external manifestations of internal processes, such as parental concerns, misinformation, and the inadequately addressed biases on vaccine safety and efficacy. For this reason, the literature contained various studies calling for the review of NME policies, especially in MMR hotspots [17,21,143].

Identifying the root causes of parental hesitancy is imperative in addressing individual concerns, particularly during physician-based vaccine discussions. Although hesitancy to the MMR vaccine has received more attention, parents were also hesitant toward other childhood vaccines such as the human papillomavirus (HPV) vaccine because of concerns for the inadvertent promotion of teen sexual activity [107,135] and the influenza vaccine for perceptions of its adverse effects [107,135]. Among ethnic groups, vaccine hesitancy was more commonly mentioned among non-Hispanic White parents [11,12,22,81], although some studies observed otherwise [83,85].

### 4.2. Themes on Vaccine Information Sources

Four sources of vaccine information were considered in this review: primary/healthcare, internet/social media, word of mouth, and print media. Regardless of vaccine views, parents considered primary/healthcare as the most trusted authorities on vaccines [31,81,122,129] with seeking a physician’s advice as a predictor of vaccine uptake [144]. Internet and social media still came in second, particularly among vaccine-hesitant parents, which implied its pervasive influence among mothers, who frequently use social media. One study stated that mothers opposed to vaccinations resorted to airing their sentiments online given the ease in posting comments [145]. Facebook, Twitter, Instagram, and YouTube served as the principal media for public communication, particularly for tech-savvy young mothers [87,112]. However, the lack of information verification and quality control with online sources can exaggerate the risk for adverse events while downplaying the benefits of vaccines.

Social media can disseminate truthful as well as misleading information. Kyle Yasuda, former president of the American Academy of Pediatrics (AAP), forged a partnership with Google, Facebook, and Pinterest to ensure that only accurate vaccine information was supported by these tech giants [144]. Despite the potential to rapidly disseminate inaccuracies, social media can be a valuable tool for countering vaccine misinformation or disinformation [146]. Healthcare providers can use social media as an educational tool, a vaccine scheduler, an appointment reminder, and an advertisement platform [87,111,112,114,140]. Polling, the number of clicks, or time spent online leave digital impressions that are trackable over time. These data can be used to assess how effectively vaccine messages reach target populations. Layering social media with the traditional radio, TV, and print outlets helps geo-target specific communities needing vaccine resources. Vaccine messages tailored toward particular concerns allow healthcare and public health professionals to connect with parents on questions that are both personal and real for them [109,134].

### 4.3. Themes on the Social Determinants of Vaccine Hesitancy and Vaccine Compliance

Vaccine hesitancy is multifactorial [11,12]. Its context-specific nature is demonstrated in the variety of underlying reasons for vaccine delay or refusal. McKee and Bohannon categorized the reasons for vaccine hesitancy into four themes: (1) religious, (2) personal/philosophical, (3) safety, and (4) need for healthcare provider-based vaccine information [147]. There was a gradient in vaccine compliance and hesitancy. What seemed as mutually exclusive views of “acceptance” versus “hesitancy” or “refusal” actually occurred as a psychosocial gradient along a spectrum of perceived benefits, barriers, severity, and susceptibility [79,81]. For instance, Gust et al. categorized vaccine-hesitant parents along a continuum: “Immunization Advocates,” “Go Along to Get Along,” “Health Advocates,” “Fence-Sitters,” and “Worrieds” [148]. Similarly, Leask et al. classified parents based on their perception of vaccine risk: “Unquestioning Acceptors,” “Cautious Acceptors,” “Hesitants,” “Late/Selective Vaccinators,” and “Refusers” [149].

Vaccine behavior is complex and multifaceted. To comprehend vaccine behavior and design interventions that will promote vaccine uptake, Brewer et al. offered three psychological propositions based on the constructs of various theories of behavior change: thoughts and feelings, social processes, and interventions that persuade but which do not change thoughts and feelings [26]. These propositions aligned with the foremost reasons for vaccine hesitancy identified by the SAGE Working Group: (a) health beliefs and attitudes, (b) perceived vaccine risks and benefits, and (c) communication and media environment [27]. According to Brewer et al., thoughts, feelings, and social norms/processes as motivators of vaccine behavior rely heavily on risk appraisal and on confidence or trust as correlates of vaccine behavior, the crucial denominator of which is risk perception—a construct common to the Health Belief Model [26], Fishbein and Ajzen’s theory of Planned Behavior [36], and Rogers’ Protection Motivation Theory [26,79,81]. Applying these theories in vaccine messaging may help counter deeply-embedded beliefs and misinformation [26,79,81].

Social factors exert influences at multiple socioecological levels—individual, family, community, and society. They determine parental vaccine perceptions and decisions. However, the direction of influence is crucial. Social factors can exert a bidirectional influence—that is, they can facilitate or hinder vaccine compliance depending on how a particular social factor is experienced. For example, physician trust, as a primary care/healthcare determinant, can encourage parental compliance to vaccination, whereas poor patient–physician communication can discourage pro-vaccine behavior. 

Education and socioeconomic status had contrasting influences on vaccine hesitancy [9]. The positive correlation between vaccine hesitancy, income, maternal education, and NMEs emerged as the predominant pattern among the articles included in this review [150]. The prevalent SDH profile of vaccine-hesitant parents included older, college-educated, high-income, married, non-Hispanic White mothers [11,12,22,23,37,58,61,70,78,81,117,122,129,134,150,151]. Conversely, some articles discussed vaccine hesitancy among mothers with less than the median annual household income, with a high school education or less, and/or coming from minority and/or immigrant populations [83]. 

No generic explanation emerged from the literature on the divergent SDH profiles of vaccine-hesitant parents as to income and maternal education. Nevertheless, some authors offered potential reasons. First, there is a socioecological interplay between parent-specific (race/ethnicity, education, income, vaccine experiences/information), vaccine-specific (perceptions of safety, efficacy, disease susceptibility), and external factors (patient–provider relationship, school immunization requirements, collective values, social norms, policies, media) [11]. Given such interplay, “philosophical and ideological” factors can drive vaccine behavior more than the “empirical knowledge” of vaccines [152]. For instance, psychosocial/behavioral determinants were noted among affluent and highly educated mothers [117]. Further, as health decision makers in the home, strong peer influence could have substantial sway on mothers’ beliefs, opinions, and intentions to delay or refuse the MMR vaccine and/or other childhood vaccines [117]. The value that these influences hold suggests the need for understanding the unique social conditions in measles hotspots to determine not only the reasons for hesitancy, but also how mothers could become positive vaccine influencers. Congruent with social learning, vaccine strategies need to integrate behavior modeling and the positive influences of family, social support networks, and trustworthy sources to reinforce trust and vaccination intent. 

Second, the sociodemographic correlates of vaccine hesitancy clustered in communities in ways that promoted vaccine hesitancy [153,154]. For example, the measles outbreak in San Diego, California, demonstrated the spatial clustering of vaccine refusal and NMEs among college-educated parents of unvaccinated kindergarteners in middle- to upper-income neighborhoods [23]. Kim [22] obtained similar results regarding the 2015 Disneyland measles outbreak: residents of cities within the southern boundaries of Orange County, California, had higher income levels, higher rates of NMEs, and were more likely to follow alternative vaccine schedules compared to those residing in the northern part of Orange County. Conversely, greater MMR and DTaP uptake was noted among those with higher income and education [47,72] compared to those who lived in low-income communities. Cataldi, Dempsey, and O’Leary observed that vaccine-hesitant mothers were less aware of the Disneyland measles outbreak, had lower income and educational status, and were less likely to have private health insurance [38].

Third, the contrasting distribution of vaccine hesitancy and NMEs as to income and education may reflect social and health inequities [11,154]. For example, Gowda and Dempsey alluded to the influence of income on interpreting common vaccine terminologies such as “vaccine safety” and “vaccine-related adverse effects” (injection-related side effects of pain, soreness, and fever) [11]. Further, the socioeconomic context of undervaccination differed from that of unvaccination. Undervaccination was typically influenced by access, cost, and continuity of care while unvaccination was primarily driven by personal beliefs and choice [154]. For example, groups of unvaccinated kindergarteners clustered in affluent neighborhoods with pro-vaccine hesitancy social norms and strong intentional philosophical views and whose healthcare resources allowed for insurance-covered physician visits for a more spread-out alternative vaccine schedule [22,23]. The parents of these kindergartners were twice as likely to be concerned about vaccine safety, had lower perceived needs for vaccination given the reliance on herd immunity, and believed that schools should allow their children entry despite being unvaccinated [22]. Undervaccinated children (vaccine schedule delays/longer time to vaccination) were from low-income households with multiple needs and competing priorities that resulted in unintentional delays rather than a deliberate choice [154]. Some studies in this review noted that parents with lower income and inadequate healthcare insurance coverage had greater concerns on vaccine safety and efficacy and were unfamiliar with recommended vaccine schedules; these families juggled family responsibilities against less flexible work schedules, particularly those who were hourly wage workers, had dual-working parents, or were single-parent households [72,87,111,115,122,141]. 

Finally, the generational shift in vaccine-hesitancy patterns through the years may explain the differential impact of education and socioeconomic status on vaccine hesitancy. Prior to the measles elimination period, vaccine hesitancy patterns in the 1980s and 1990s were largely unintentional and was associated with poverty and lack of vaccine access [155], particularly among immigrant families with limited education [156]. On the other hand, articles in this review were published between 2000 and 2022 and highlighted the positive correlation between vaccine hesitancy, income, education, and NMEs—the timing of which corresponded with greater information availability and access through the internet and social media, which can influence mothers’ decisions on their children’s vaccination status.

Vaccine holdouts are geographically spread out across the United States. Spatial clusters were noted largely in the West (California, Washington, Oregon, Colorado, Utah, and Arizona) with some in the Midwest (Missouri, Illinois, Indiana) and Northeast [12,22,23,37,38,46,47,49,58,59,60,61,71,78,79,83,98,123,129,134]. However, the urban–rural classification of these communities was not specified. The United States Health Resources and Services Administration’s (HR SA) 2019 article on measles vaccine hesitancy, which was not part of this review, addressed this gap in the data by listing the urban hotspots of vaccine resistance based on reported rates of NMEs: Seattle, Spokane, Portland, Phoenix, Salt Lake City, Houston, Fort Worth, Austin, Detroit, Kansas City, smaller counties in Indiana, Wisconsin, and Utah, and eight counties in Idaho [145]. Comparable results were obtained by Gardner et al., who conducted a spatial analysis to illustrate the geographic increase in MMR-susceptible communities in densely populated urban areas based on MMR vaccination rates, county population, volume of international travel to a United States county, and incidence rates of measles from countries of origin, particularly from New Zealand, Philippines, Samoa, and Ukraine [157]. Gardner et al.’s spatial model showed that California (Los Angeles, Santa Ana), New York, Washington (Seattle), Texas, and Florida (Miami) have the highest risk for measles outbreaks—areas that corresponded with the actual 2019 surge in measles in 31 states in the country [8].

### 4.4. Themes on Primary Care/Healthcare Strategies

Crucial to the parent–physician interaction are trust and respect—the same foundational elements of vaccine confidence. Allowing individuals and parents to make an informed choice by providing them with the best available scientific information without evading questions on the adverse effects of vaccines will strengthen the belief that healthcare providers are looking out for their welfare [12,108,112].

Based on the literature, the key to effective messaging was identifying vaccine sources and/or messengers that are perceived to be trustworthy. Yet, physicians, as trusted messengers, found it challenging to convey vaccine information to those with strongly held views or who were resistant to further information [112,134]. 

Articles in this review stressed the value of training physicians on handling difficult conversations with vaccine-hesitant parents. AAP’s Committee on Bioethics recommended against discontinuing the care for patients who declined vaccines; instead, it believes in continuing the care for parents/families with opposing vaccine viewpoints [129].

Parental confidence on vaccines is largely influenced by how compellingly vaccine recommendations are communicated by healthcare providers. Studies showed that parents who decided to have their children vaccinated were more likely to have been positively influenced by the examples and strength of conviction of their physicians regarding the safety and efficacy of vaccines [11,150]. Nevertheless, articles in this review advised caution on physicians sharing their own vaccine questions with their patients during consultations since these may be misconstrued as an expression of doubt, even resistance, to certain vaccines [79].

The literature was replete with recommendations that were applicable in the clinical setting. The most commonly emphasized suggestion was enhancing parent–physician trust by listening with empathy in purposeful, open, respectful, empathetic, non-judgmental, and unrushed dialogues on vaccines [62,113,123,129]. Parents preferred longer interactions with their physicians to openly discuss their questions without fear of criticism or judgment [71,106]. Although certain vaccine concerns could take more than one visit, recurring discussions may help educate parents on the benefits of vaccines while uncovering deeply entrenched reasons for hesitancy [144]. Other articles suggested addressing the specific barriers to vaccination and providing financial incentives to healthcare providers who invest time in advising vaccine-hesitant parents [12,106]. There was also a repeated emphasis on having healthcare providers stay up-to-date on the latest vaccine safety information [129,130]. A robust knowledge of vaccines would allow for detailed conversations that could ideally start during prenatal visits [130,135]. Such dialogues could win over current and future mothers, thus allowing positive vaccine perspectives and behavior to be nurtured and passed on intergenerationally in families and households. 

Articles included in this review called for collaborative efforts between healthcare providers and the community to fight vaccine misinformation. Crafting clear, accurate, and compelling vaccine messages is vital in communicating with parents, particularly in addressing the misinformation and concern on the purported link between autism and the MMR vaccine [35,39,57,76,85,86,93,97,137]. Science alone has not been fully effective in eliminating parental fears of autism [76]. The pervasiveness of this concern calls for alternative interventions to complement the dissemination of information on rigorous population-based vaccine studies that debunked this link [93]. In addition to social factors, vaccine hesitancy toward MMR is influenced by having children with developmental conditions such as Autism Spectrum Disorder (ASD) [57]. To address the confusion on the causes of ASD and to debunk the belief that toxins in vaccines cause ASD, clear communication is necessary using different platforms (social media, medical, governmental, and educational) to curb the spread of misinformation [57,76]. Open conversations with physicians, particularly developmental pediatricians, may dispel the misinformation about autism and MMR [57,76]. Prospective, longitudinal studies are likewise vital in determining when and how parental vaccine concerns emerged in an ASD-vaccine scenario, the findings of which can serve as a basis for clinical and public health education [35]. 

The fear of autism is common among multicultural and immigrant communities. To help build trust, correct misinformation, and mollify fears of autism in these communities, studies included in this review cited the following recommendations: (1) using competent interpreters during clinic visits, (2) providing ample time with physicians to openly discuss vaccine concerns, (3) scheduling families routinely with the same physician, (4) providing vaccine information before clinic visits, (5) using images instead of text narratives, (6) offering clear vaccine recommendations, and (7) starting the MMR discussion during the 6-month and 9-month well-child visits to mentally prepare parents on the value of timely vaccinations [85,86]. Involving community figures, such as imams in Somali migrant communities, to advise public health practitioners on culturally appropriate vaccine initiatives may also be beneficial [97]. Cultural practices, such as the strong oral tradition among Somalis, can be utilized in vaccine efforts to positively influence parental peer networks toward vaccine compliance [97]. Given the susceptibility of migrant communities to vaccine hesitancy because of trust issues and the fear of autism, Tankwanchi, et al. [137] strongly recommended that community-based vaccine delivery strategies need to also address the underlying unmet social needs through policies that protect and support the rights and dignity of migrant communities.

Other approaches mentioned in this review that could boost vaccine acceptance included the following:

- Creating innovative reminder tools such as using social media for appointments and vaccine reminders [72,80,87,141];
- Administering educational interventions in clinical settings by handing out vaccine information to patients in the waiting room [104,106,139,140];
- Connecting emotionally with parents by having physicians share positive personal vaccine stories, family experiences, and personal narratives that are understandable and memorable [106,109,140];
- Utilizing visually enhanced education (VEE) techniques such as pictures, storyboards, or videos on vaccine-preventable diseases (VPDs) to educate parents on the serious health complications of VPDs [72,100,130,139];
- Using simple, clear, and succinct language to convey scientific information by using fewer jargon and clinical explanations and more of simple descriptions or analogies [104,113,119,120];
- Composing relatable and easily understood metaphors in discussing vaccine safety, benefits, and adverse effects [106];
- Applying motivational interviewing techniques using the “Plan, Do, Study, Act” (PDSA) or the “corroboration, about me, science, explain” (CASE) methods to understand deeply held reasons for hesitancy or refusal [57,119,120,121,136,139];
- Using a presumptive tone rather than a participatory tone (“We will do the shots” versus “What do you want to do about the shots?” conveys the provider’s confidence in vaccines and establishes vaccines as a routine part of a well-child visit) [109,112,121,135,139];
- Applying evidence-based pain control strategies to reduce fear of injections [135].

### 4.5. Themes on Public Health and Government-Level Strategies

Mistrust in government and pharmaceutical companies was another reason for vaccine hesitancy (see Table 2). Vaccine mandates were viewed by some as an infringement of personal, parental, or constitutional rights, which has incited further resentment and distrust [59,109,112]. With the strong sentiments over individual rights versus government-initiated vaccine mandates, balancing personal autonomy with the public’s safety remains at the crux of vaccine debates, discussions, and decisions. Vaccine mandates largely focused on increasing vaccine coverage, but they did not address the roots of parental vaccine hesitancy. Although policy restrictions and monetary sanctions could increase vaccination rates, articles in this review emphasized preserving the relationship of trust between parents and physicians. 

Social factors influence NMEs. In this review, the increase in NMEs was facilitated by lenient state and/or county exemption policies, resulting in geographically concentrated pockets of susceptible individuals [21,158]. Articles in this review called for legislative action to reexamine NME policies, particularly in communities at risk for outbreaks. Eliminating NME policies has been shown to increase MMR vaccine coverage [21]. However, stringent policies could have unforeseen blowbacks [159,160]. For instance, the state of California passed Senate Bill No. 277 (SB-277) in 2015 to prevent future measles outbreaks by prohibiting the admission of any unvaccinated child in “public or private elementary or secondary school, child care center, day nursery, nursery school, family day care home, or development center” until all required immunizations are completed, including those against measles and pertussis [161]. SB-277 eliminated personal or religious-belief-based exemptions but allowed for medical exemptions if accompanied by a physician signature, a policy similarly required by Washington State [21,161]. Although vaccination rates increased among kindergarteners across the state, it inadvertently led to the rise in medical exemptions in just three years after the enactment of the bill [162]. Medical exemptions more than quadrupled from 0.2% in 2014–2015 to 0.9% in 2018–2019 [163]. By 2017, more than 10% of kindergarteners in 58 schools were unvaccinated because of medical exemptions while seven schools had more than 20% exemptions based on a *Los Angeles Times* analysis [164]. These exemptions were concentrated in Los Angeles County, San Diego, and Orange County [164]. After the passage of SB-277, websites appeared with detailed instructions on how to request for medical exemptions, complete with a list of physicians that parents could approach to certify such exemptions. To curtail such consequence, Governor Gavin Newson signed SB-276 into law on 9 September 2019, which took effect on 1 January 2021 [165]. SB-276 required the California Department of Public Health to oversee, approve, and standardize applications for medical exemptions that meet the CDC/Advisory Committee on Immunization Practices (ACIP) or AAP criteria [165]. Experts emphasized that the key is balancing the toughness of a policy with public health efforts to educate parents [21,165]. States such as Oregon require parents to watch an educational module prior to obtaining NMEs [21]. 

NMEs require methodical policy review and foresight. Even a small reduction in herd immunity because of NMEs could cause serious measles outbreaks. A stochastic mathematical modeling by Lo and Hotez showed that a 5% dip in MMR vaccination coverage could mean a three-fold rise in measles cases nationally among children from two to eleven years of age with a corresponding escalation in health expenditures by another $2.1 million [166]. Given the significant impact of such outbreaks on the nation’s health and economy, Lo and Hotez called for the removal of NMEs.

Addressing vaccine hesitancy requires a multisectoral and multimodal approach. Collaborations between healthcare providers, public health practitioners, vaccine manufacturers, and policymakers are crucial in combating misinformation, verifying reported vaccine adverse effects, coordinating system-level vaccine logistics, and creating innovative pro-vaccine strategies [144]. Partnerships between the government and pharmaceutical companies are vital in informing the public about the rigorous vaccine development and safety monitoring process—from research, testing, approval, manufacturing, scheduling, deployment to continuous surveillance [167]. None of the articles in this review assessed parental awareness of the ongoing CDC and FDA vaccine monitoring following vaccine deployment. For instance, the Vaccine Adverse Event Reporting System (VAERS) collects and analyzes reports from parents, patients, or healthcare providers [167]. The Vaccine Safety Datalink (VSD) and Post-Licensure Rapid Immunization Safety Monitoring (RISM) are networks of US healthcare organizations that analyze the clinical information of over 24 million and over 190 million people, respectively [167]. Additionally, the Clinical Immunization Safety Assessment (CISA) Project, a CDC and medical research center collaboration, conducts continuing clinical studies on vaccine safety and adverse events [167].

### 4.6. Strengths and Limitations

Systematic reviews often search for relevant literature in two or three databases. However, our study relied on seven large citation databases for identifying potentially eligible studies, which is a strength of our study. We selected databases that would allow us to include a comprehensive mix of quantitative, mix-methods, and qualitative studies that met our inclusion criteria. We added qualitative studies for a fuller and richer identification and analysis of parental vaccine perceptions and how social variables influenced vaccine beliefs and decision-making, particularly toward MMR. Nevertheless, it is possible that we missed relevant studies that met our inclusion criteria, but which were not cited in the databases that we selected. In addition, publication bias—or the preference toward studies with statistically significant results—may have also influenced the studies cited and eventually included in this systematic review. 

Although we added academic theses and dissertations, we did not include other gray literature documents, such as government reports, proceedings, white papers, and newsletters. These could have identified current parental reasons for MMR vaccine hesitancy, including perspectives that were not previously mentioned in published studies. 

We assessed the quality of quantitative, mix-methods, and qualitative studies using the Mixed Methods Appraisal Tool (MMAT) [30]. Although 77% of the included studies satisfied all five methodological quality criteria, inherent variations in the aims and study designs of the included studies complicated our analysis. For instance, the included studies differed as to aims, study settings, subject demographics, and analyses. Some included studies did not fully report or contextualized the demographic characteristics of participants.

The included studies were published from 2000 to 2022—from the year that measles was eliminated in the US to the occurrence of the COVID-19 pandemic. The more than two-decade span of time allowed for more studies to be included in this review. However, vaccine sensibilities could have changed significantly through time as influenced by the prevailing social, health, economic, political, and environmental issues, especially with greater connectivity through the wider use of and access to technology, including social media. Although we noted similarities in parental perspectives to MMR and other childhood vaccines in more than two decades of published research, it is still possible that there were generation-specific vaccine issues that influenced parental vaccine sensibilities that we did not explore in this study. In addition, infectious disease outbreaks, such as the COVID-19 pandemic, can influence parental health behavior. The underlying reasons for the change in vaccine perspectives through time—at the population, community, family, and parental levels—could be helpful for informing and crafting effective and up-to-date vaccine messaging, especially regarding MMR. Since systematic reviews utilize a retrospective, observational research design, these are subject to several limitations and so is our study. Despite our best efforts to reduce bias between coders, it is still possible that there was inconsistency and selectivity in reporting the findings of included studies.

## 5. Conclusions

Vaccine hesitancy is a public health threat. It undermines historical achievements and thwarts years of progress in the fight against infectious diseases. It is marked by low parental vaccine trust, poor vaccination uptake, and high NMEs. The geographic clustering of vaccine hesitancy, particularly against the MMR vaccine, indicates that social drivers shape parental perceptions and decisions on immunization. 

The findings of this review may have potential implications in tackling hesitancy toward other vaccines. Geographic clusters of under- and unvaccinated individuals where NMEs predominate and where social determinants facilitate vaccine hesitancy could serve as hotspots for other infectious diseases. As the United States and the world continue to confront the resurgence of vaccine-preventable diseases and the emergence of new infections, and current and future pandemics, it becomes imperative to develop strategies that target the social conditions that drive vaccine hesitancy. Additional empirical studies are needed to test such strategies and to determine how social factors explicitly impact parental vaccine views, attitudes, and behavior. 

Addressing vaccine hesitancy is a collective responsibility. A one-size-fits-all approach is unlikely to be successful. Nurturing partnerships of trust among parents, physicians, and government sectors is crucial in dispelling myths and doubts on the benefits and safety of vaccines. Policymakers can start with examining what drives NMEs. Healthcare providers and public health practitioners can explore innovative and culturally appropriate ways of reaching out to parents, regardless of vaccine beliefs. Engaging parents in safe and nonjudgmental discussions is vital in effectively tackling vaccine misinformation and hesitancy. Combining the science of vaccines with an in-depth understanding of parental sentiments could open up conversations among those with lingering concerns. Using messages tailored to specific issues may improve the vaccination rates among traditionally hesitant populations. 

The family can be an important public health ally in expanding vaccine acceptance. Implementing family-centered approaches may help reinforce the social determinants that promote parental assent. A glaring gap in the literature is the role of fathers in vaccination decisions. Similarly, the involvement of the whole family unit in curbing vaccine hesitancy has not been adequately researched. Studies in the literature focused primarily on mothers. Although this serves as a tacit recognition of the role of mothers as health decision makers in the home, it ignores the potential of fathers—and the whole family unit—to balance perspectives and counter the barrage of negative vaccine messages. Moreover, vaccine ideologies and decisions can be transmitted intergenerationally. Grandparents, as informal caregivers, can serve as trusted messengers whose positive personal experiences and stories on vaccine-preventable diseases can help counter misinformation and medical mistrust while also providing multi-generational support to the family.

## Figures and Tables

**Table 1:** Summary on vaccine hesitancy and parental demographic variables.

Demographic Variable and Total Number of Articles per Variable	Relationships Mentioned or Observed in Articles	Number of Articles	Summary
Parental Age (15 articles)	Older parental age and higher vaccine hesitancy	7	Several articles included in the review noted higher vaccine hesitancy among older parents (≥30 years of age) in association with education, income, marital status, and number of children.
	Younger parental age and higher vaccine hesitancy	4	
	No relationship observed between parental age and vaccine hesitancy	4	
Parental Race/Ethnicity (30 articles)	Non-Hispanic White race and higher vaccine hesitancy	15	Several articles included in the review cited higher vaccine hesitancy and vaccine refusal among non-Hispanic White parents. Unvaccinated or undervaccinated children were more likely to be White and/or have parents with NMEs *.
	Non-White race and higher vaccine hesitancy	11	
	Other race/ethnicity findings	4	
Education (39 articles)	Higher level of parental education and higher vaccine hesitancy	17	Contrasting patterns were noted as to level of education. Several articles included in the review noted higher vaccine hesitancy among parents with college education or higher. Almost the same number of articles mentioned higher vaccine hesitancy among parents with lower education. Vaccine hesitant parents were more likely to enroll their children in private, charter, or home-based schools.
	Lower level of parental education and higher vaccine hesitancy	15	
	No relationship observed between level of parental education and vaccine hesitancy	4	
	Higher level of parental education and lower vaccine hesitancy	1	
	Private, charter, or home-based schools and higher vaccine hesitancy	2	
Income/Socioeconomic Status (SES) (26 articles)	Higher SES/income and higher vaccine hesitancy	12	Contrasting patterns were noted as to income/socio-economic status. An equal number of articles included in the review noted vaccine hesitancy in association with both higher and lower income/SES.
	Lower SES/income and higher vaccine hesitancy	11	
	No relationship observed between SES/income and vaccine hesitancy	3	
Health Insurance (5 articles)	Health or private insurance and vaccine hesitancy	3	Having health insurance and/or the type of health insurance influenced vaccine hesitancy.
	Public/lack of health insurance and vaccine hesitancy	2	
Social Influences/Social Network (2 articles)	Place-based ideological sorting as to socio-economic status, political affiliations, or religious beliefs and higher vaccine hesitancy	1	Social networks and geographical patterns in ideological clustering of NMEs influenced vaccine hesitancy.
	Local spread of vaccine beliefs via parental social networks and higher vaccine hesitancy	1	
Policies/Political Affiliation (10 articles)	Policies (ex. SB277, NMEs, vaccine policies) Parents who identified as Democrat and higher vaccine hesitancy	8	Policies (NMEs and vaccine-specific policies) influenced vaccine hesitancy. Higher vaccine hesitancy was noted among parents who filed for NMEs.
	Parents who identified as Republican/Conservatives and vaccine hesitancy	1	
	Parents who identified as neither Democrat nor Republican and vaccine hesitancy	1	
Religion/Religiosity (5 articles)	Higher religiosity/religious objections and higher vaccine hesitancy	3	Religious beliefs and objections influenced vaccine hesitancy and outbreaks in measles cases.
	Ultra-Orthodox Jewish communities in New York and higher vaccine hesitancy	2	
Urban/Rural Areas (2 articles)	Residing in non-metropolitan statistical areas (MSA) or rural areas and vaccine hesitancy	1	Residing in rural vs. urban areas influenced vaccine hesitancy. Residing in rural areas and lack of insurance were associated with undervaccination.
	Residing in certain MSA and in rural areas and vaccine hesitancy	1	
Chronic Conditions (3 articles)	Parents of children with Autism Spectrum Disorder (ASD) or Down Syndrome and higher vaccine hesitancy	3	Being a parent with a child diagnosed with Autism Spectrum Disorder or Down Syndrome influenced vaccine hesitancy.
Marital Status (9 articles)	Married parents and higher vaccine hesitancy	7	Higher vaccine hesitancy was noted among married vs. single parents.
	Single parents and higher vaccine hesitancy	2	

* Non-medical exemptions.

**Table 2:** Parental vaccine concerns identified in the literature (frequency of themes mentioned out of 115 articles reviewed).

Theme	Belief	General Vaccines *f* (%)	MMR ^†^ Specific *f* (%)
Danger/risk	Vaccine adverse reactions/hypersensitivity reactions	86 (75)	36 (31)
	General/other safety concerns	54 (47)	18 (16)
	Risk of autism	49 (43)	62 (54)
	Overwhelms immune system	37 (32)	1 (1)
	Concerns with vaccine components	33 (29)	13 (11)
	Pain on injection site	26 (23)	6 (5)
Personal concern	Too many vaccines/concerns with vaccine schedule	49 (43)	9 (8)
	Mistrust of government and health officials	45 (39)	9 (8)
	Preference for natural immunity	31 (27)	3 (3)
	Philosophical/moral objection	27 (23)	5 (4)
	Cost or access to vaccines	26 (23)	6 (5)
	Religious opposition	24 (21)	8 (7)
Perceived benefits	Low perceived benefit/susceptibility	45 (39)	14 (12)
	Vaccine efficacy concerns	26 (23)	8 (7)
	Not recommended by healthcare provider	7 (6)	0 (0)

^†^ Measles, mumps, and rubella vaccine.

**Table 3:** Most commonly used vaccine information sources based on parental vaccine views (frequency of themes mentioned out of 115 articles reviewed).

Source	Parent Category		
	Hesitant *f* (%)	Compliant *f* (%)	Not Specified *f* (%)
Healthcare sources	44 (38)	56 (49)	68 (59)
Internet/social media	52 (45)	25 (22)	52 (45)
Word of mouth	37 (32)	18 (16)	40 (35)
Print/broadcast media	21 (18)	15 (13)	25 (22)

**Table 4:** Most trusted vaccine information sources based on parent vaccine views (frequency of themes mentioned out of 115 articles reviewed).

Source	Parent Category		
	Hesitant *f* (%)	Compliant *f* (%)	Not Specified *f* (%)
Healthcare sources	31 (27)	46 (40)	45 (39)
Internet/social media	16 (14)	6 (5)	9 (8)
Word of mouth	15 (13)	5 (4)	6 (5)
Print/broadcast media	4 (3)	2 (2)	4 (3)

**Table 5:** Social determinants of health identified as facilitating or hindering parental vaccine compliance (frequency of themes mentioned out of 115 articles reviewed).

Social Determinant	Facilitating *f* (%)	Hindering *f* (%)
Primary care/healthcare	46 (40)	38 (33)
Education	30 (26)	38 (33)
Government/political	21 (18)	26 (23)
Psychosocial/behavioral	20 (17)	47 (41)
Public health	19 (17)	17 (15)
Economy/income	18 (16)	30 (26)
Environment/built environment	4 (3)	9 (8)
Housing	4 (3)	6 (5)
Employment	2 (2)	3 (3)
Transportation	2 (2)	4 (3)

## Data Availability

Not applicable.

## Supplementary Materials

The following supporting information can be downloaded at: https://www.mdpi.com/article/10.3390/vaccines11050926/s1, Figure S1: Systematic Review Codebook, Table S1: Systematic Review Search Terms and Databases, Table S2: MMAT Assessment of Methodological Quality, Table S3: Characteristics of Studies Included in the Systematic Review, Table S4: Vaccine Hesitancy and Parental Demographic Variables.
