# Supplementary material for: Why Parents Say No to Having Their Children Vaccinated against Measles: A Systematic Review of the Social Determinants of Parental Perceptions on MMR Vaccine Hesitancy
Source: Vaccines (Basel). 2023 May 2;11(5):926. doi: 10.3390/vaccines11050926 (PMC10224336; doi:10.3390/vaccines11050926)
Supplement: Supplementary file 1 [file vaccines-11-00926-s001.zip › Figure S1 Systematic review codebook.pdf]

Figure S1: Systematic Review Codebook

Research Questions

1. Why do parents delay, refuse, or oppose to having their children vaccinated against measles?
2. What are the parental perceptions, attitudes , and practices on measles vaccination?
3. Where do parents/caregivers obtain measles vaccine information? Which source do parents/caregivers trust on measles immunization?
4. Which social factors impact parental perceptions, attitudes, and practices promote or hinder measles vaccine hesitancy?
5. How can families, primary care, health care, public health, and government address measles vaccine hesitancy?

Methods: Qualitative Systematic Review

According to the Cochrane Review, "a systematic review attempts to identify, appraise and synthesize all the empirical evidence that meets pre-specified eligibility criteria to answer a specific research question. Researchers conducting systematic reviews use explicit, systematic methods that are selected with a view aimed at minimizing bias, to produce more reliable findings to inform decision making."

A systematic review is completed in the following steps:

1. Identify relevant studies from a number of different sources (including unpublished sources);
2. Select studies for inclusion and evaluation of their strengths and limitations on the basis of clear, predefined criteria;
3. Collect data systematically;
4. Synthesize the data

As PI and Co-PI, we have completed the following initial steps:

1. Create the inclusion criteria and search words;
2. Identify relevant articles/studies in the literature using the search words;
3. Complete the abstract review;
4. Develop the Codebook; and
5. Prepare the coding in Qualtrics.

From the initial search, we gathered 421 articles for the abstract review. We have narrowed these down to195 articles for the full text review.

As coders, you will assist in conducting the full text review. This will entail careful reading and review of each article. This will be followed by coding the article for specific information that answers our various research questions. To have consistency and agreement between coders, we will conduct several rounds of preliminary coding or test coding by reading a random list of 10 or 15 articles. After completing each round of individual test coding, as a team, we will discuss how each article was coded to determine consistency in coding. Where there are discrepancies in coding a certain article(s), as a team, we will code it together based on the Codebook.

Coding Instructions:

Note on Preliminary Coding/Test Coding: This step determines where we are similar or different in our coding. Our goal is to be consistent in our coding across all coders. For example: Did you code article #3 in the same way that I coded it? Did I code article #3 in the same way you did?

1. Carefully read the full text of each article included for systematic review.
2. Code the article for specific information (classify and/or summarize) based on the research questions by opening a data extraction form in Qualtrics. This link opens up a set of questions regarding the article that you have read based on our research questions.
3. Complete the Qualtrics data extraction form for each article as accurately as possible.
4. Repeat the process for the next article.
5. Meet as a team after individually completing the preliminary coding on Qualtrics. As a team, we will identify, discuss, and reconcile any disparities in the coding of each article. We will also identify common errors in coding to avoid these errors prior to the full-text review. We will repeat this process until we have reached 100% consensus on the coded articles.

Inclusion Criteria

- **English language articles**

- **Publication year**
  0 – 1999 and earlier
  1 – 2000 to 2019

- **Empirical research studies and literature reviews** *(journal articles, dissertations, and theses)*

- **Place of study**
  0 – Outside the U.S.
  1 – U.S. and/or any U.S. states mentioned

- **Content 1: Measles /MMR Vaccination**
  0 – Does NOT discuss or mention the measles/MMR vaccine/vaccination
  1 – Discusses measles/MMR vaccine/vaccination
  2 – Discusses other vaccines and measles/MMR vaccine/vaccination

- **Content 2: Parents'/Caregivers' views, beliefs, attitudes, or practices on the measles /MMR vaccine/vaccination (vaccine hesitancy, refusal, opposition)**
  0 – Does NOT mention/contain information on parents'/caregivers' views, beliefs, attitudes, or practices on measles/MMR vaccine/vaccination
  1 – Discusses/mentions parents'/caregivers' views, beliefs, attitudes, or practices on measles/MMR vaccine/vaccination
    A. Hesitancy, refusal, opposition to measles/MMR only
    B. Hesitancy, refusal, opposition to other vaccines but NOT to measles/MMR
    C. Hesitancy, refusal, opposition to all vaccines

- **Reason for Vaccine Hesitancy, Refusal, or Opposition:**
  0 – Not mentioned
  1 – Cost or access to vaccines
  2 – Pain on injection site
  3 – Vaccine adverse reactions including hypersensitivity
  4 – Others

- **Classification of parents/caregivers:**
  0 – Not mentioned
  1 – Mother
  2 – Father
  3 – Grandparents
  4 – Relatives taking care of child
  5 – Caregiver (not biologically-related to child)

- **Social determinants of parents'/caregivers' views, beliefs, attitudes, or practices**
  0 – Does **NOT** discuss/mention any social factors associated with vaccine views, beliefs, attitudes, or practices on the measles vaccine/vaccination
  1 – Discusses/mentions any social factors associated with vaccine views, beliefs, attitudes, or practices on measles/MMR vaccine/vaccination
    A. Parent's/Caregiver's Age: *(Indicate if mentioned)*
    B. Parents'/Caregivers' Race/Ethnicity: *(Indicate if mentioned)*
    C. Parent's/Caregiver's Level of Education:
      C1 – Less than highs school
      C2 – High school graduate
      C3 – Some college
      C4 – College graduate
      C5 – Graduate level
      C6 – Doctorate or professional degree
  D. Parent's/Caregiver's Household Income
      D1 – Below poverty level
      D2 – At poverty level
      D3 – Median level
      D4 – High level
  E. Parent's/Caregiver's Health Insurance
      E1 – Private
      E2 – Employment-based
      E3 – Medicaid, etc.
  F. Parent/Caregiver Home Ownership
      F1 – Owns home
      F2 – Rents
      F3 – Lives with relatives
  G. Political Affiliation
      G1 – Democrat
      G2 – Republican
      G3 – Other
      G4 – Not mentioned

- **Source of vaccine information:** Where parents/caregivers are getting their information regarding the measles/MMR vaccines and other vaccines
  1. – Physicians, nurses, primary care clinic/healthcare
  2. – Local county health department
  3. – Schools
  4. – Social media: blogs, websites, discussion forums, or any reference to the following:
     - Social Networks: Facebook, Twitter, LinkedIn
     - Media Sharing Networks: Instagram, Snapchat, YouTube
     - Discussion Forums: Reddit, Quora, Digg
     - Bookmarking & Content Curation Networks: Pinterest, Flipboard
     - Consumer Review Networks: Yelp, Zomato, TripAdvisor
  5. – Television
  6. – Radio
  7. – Print media: printed communication from newspapers, magazines, newsletters, outdoor billboards, transit posters, yellow pages, direct mail
  8. – Word-of-mouth: oral communication with relatives, friends, neighbors, church, clubs, organizations, etc.

- **Factors that facilitate or hinder measles immunization**
  1. – Hinder: *Briefly describe factor(s) or use actual words from the article using quotation marks*
  2. – Facilitate/promote: *Briefly describe factor(s) or use actual words from the article using quotation marks*

- **Type of study**
  1. – Quantitative: collecting and analyzing numerical data to test hypotheses, describe characteristics, or determine associations/correlations; ex: survey, descriptive, correlational, experimental studies

  2. – Qualitative: collecting and analyzing non-numerical data; ex: Descriptive study, conceptual paper, interview/in-depth interview, focus group, ethnography, oral history, case study, narrative, history, use of phenomenological method ("lived experiences" by collecting data from individuals and

describing each experience) or grounded theory (comparing and analyzing data from a variety of sources)

3. – Mixed Methods Analysis: combining qualitative and quantitative methods into a single study

- **Content 3: Approaches to addressing vaccine hesitancy, refusal, or opposition among parents/caregivers**. Mentioned, listed, and/or described recommendations and/or strategies for addressing vaccine hesitancy at the primary care/health care, public health, and government levels.
  1. – Role of family: *(List and describe)*
  2. – Role of primary care physicians: *(List and describe)*
  3. – Role of public health: *(List and describe)*
  4. – Role of healthcare organizations: *(List and describe)*
  5. – Role of government: *(List and describe)*

Coding of the Social Determinants of Health

Reference: SDH Directory by Hillemeier et al.

- Data Set Directory of Social Determinants of Health at the Local Level. Hillemeier M, Lynch J, Harper S, Casper M. Data Set Directory of Social Determinants of Health at the Local Level. Atlanta: U.S. Department of Health and Human Services, Centers for Disease Control and Prevention; 2004. Available at: https://www.cdc.gov/dhdsp/docs/data_set_directory.pdf

Original Dimensions of the Social Environment based on Hillemeier et al:

1. Economy
2. Employment
3. Education
4. Political* *(Note: combined with "Governmental" in the review)*
5. Environmental
6. Housing
7. Medical/Healthcare
8. Governmental*
9. Public Health
10. Psychosocial***(Note: combined with "Behavioral" in the review)*
11. Behavioral
12. Transport

Social Factors Included in the MMR Vaccine Hesitancy Study

In coding the articles, we are looking specifically for the social determinants that may impact vaccine hesitancy. We are defining each of these social determinants based on the components and/or indicators used by Hillemeier et al. to describe each social dimension. Code any mentioned social factor(s) in each article based on how closely it meets the definitions listed below:

1. Economy & Income

- Income *(Earned income, disposable income, income distribution)*
- Wealth *(Geographic concentration of wealth, debt levels, savings rates, real estate ownership/values)*
- Poverty *(Geographic concentration of poverty, deprivation associated with poverty-level income)*
- Economic Development *(Productivity, industrial mix, business lending indicators)*
- Financial Services *(Availability of credit, availability of banking and check-cashing services)*
- Cost of Living *(Local cost of living indices, spending/consumption patterns, income-to-spending ratios)*
- Redistribution *(Taxes, transfer payments)*
- Fiscal Capacity *(Property values, sales levels, income capacity)*

2. Employment

- Employment/Unemployment Rates *(Job security, labor market turnover)*
- Workforce Characteristics *(Racial & gender diversity, skill level, unionization)*
- Area Business Capacity *(Tax breaks offered, number and size of business)*
- Job Access Occupational Safety
- Job Quality, Job Characteristics

3. Education

- Educational Attainment *(Graduation rates, dropout rates, literacy rates)*
- Funding, Private Schools *(Test scores, rates of progression to post-secondary education)*
- School Characteristics *(Size of schools/classes, student/teacher ratios, teacher turnover, parental attitude/involvement in schools, school segregation, curriculum quality, pre-school/kindergarten/early intervention, school-based clinics, physical environment of school/safety)*
- Community Climate *(Television viewing, radio stations, reading/reading to children, libraries)*

4. Governmental & Political

- Funding *(Revenue, taxes, debt)*
- Policy/Legislation *(Obstacles to unionization, living wage/minimum wage rate)*
- Services (Privatization, local services/safety net resources)
- Municipal Fragmentation *(Number of local governments, metropolitan power diffusion index)*
- Civic Participation *(Voting, census participation, political party membership, donation to parties and candidates)*
- Political Structure *(Gender, race, ethnic representation in elected office, percent of local budget for public health investments)*
- Power Groups *(Community organizations, unions)*

5. Environmental & Built-in Environment

- Air Quality
- Water Quality
- Environmental Hazards
- Physical Safety
- Land Use

6. **Housing**
   - Housing Stock *(Age of housing units, scarcity, value, characteristics, gentrification/gatedness, rental vs, owner occupied)*
   - Residential Patterns *(Homelessness, number of institutional facilities, segregation, vacancy rates, crowded housing, population density)*
   - Regulation *(Zoning policies, industrial/residential segregation)*
   - Financial Issues *(Housing costs, low-income housing, mortgage lending practices by race/ethnicity, community re-investment initiatives)*

7. **Medical/Healthcare**
   - Primary Care *(Number of providers, provider training/competence/ certification, Medicaid/Medicare reimbursement levels*); For the systematic review: may refer to nurses and/or school nurses, pediatrician(s), family physician(s), or primary care physician(s) in an outpatient clinical setting vs. hospital, ED, or tertiary level setting. Primary care is defined as the first interaction with the healthcare process (See Alma Ata and WHO definitions of primary care at (Alma Ata, 1978 at https://www.who.int/teams/social-determinants-of-health/declaration-of-alma-ata and WHO at https://www.who.int/news-room/fact-sheets/detail/primary-health-care)
   - Specialty Care *(Number of providers, Provider training/competence/ certification)*
   - Emergency Services *(Number of nonfederal physicians in emergency medicine patient care; number of hospitals with emergency departments)*
   - Home Health Care Services *(Number of hospitals with home health services)*
   - Mental Health Care *(Total number of nonfederal physicians in psychiatric, office-based patient care; number of hospitals with psychiatric emergency, outpatient, emergency social work, and outpatient social work services)*
   - Long-Term Care *(Number of nursing and board-and care homes and beds; number of long-term hospitals and beds)*
   - Oral Health Care *(Total number of active dentists in private practice)*
   - Access to/Utilization of Care *(Insurance coverage, Race/ethnicity staff-to-population ratios, Provision of care in total and indigent care, costs of care, rates of ambulatory care sensitive hospitalizations)*

8. **Public Health**
   - Programs *(Screening, Nutrition, Family planning, Chronic Disease Control, Home visiting, Outreach, School-based clinics, substance abuse prevention, domestic violence program, mental health services, immunization)*
   - Regulation/Enforcement *(Sanitation, Health/food inspection/ health violations)*
   - Funding *(Budget allocations-local health department expenditures, government expenditures on health; private sector provision of public health services)*

9. **Psychosocial & Behavioral**
   - Psychosocial
     - Political *(Contributions to parties, candidates, women in elected office, registered voters)*
     - Volunteer Organizations *(Number of churches by denomination, number of members)*
     - Union Participation
     - Charitable Giving *(Charitable contributions)*
     - Jails *(Expenditures, incarceration rates, crime)*
     - Lawsuits
     - Protective Services *(Police protection)*
   - Behavioral
     - Tobacco Use *(Smoking rates, cessation programs, smoking prevention, workplace/public space smoking restriction laws, cost/accessibility of cigarettes)*
     - Physical Activity *(Physical activity levels, physical education requirements, public and private recreational facilities)*
     - Diet/Obesity *(Fresh fruit & vegetable consumption, high-fat/high-sugar food consumption, food quality/availability, number of fast-food establishments, school nutrition)*
     - Alcohol and Illicit Drug Use *(Number of beer, wine, liquor stores, drug and alcohol treatment services, syringe laws/exchange programs)*
     - Violence *(Guns, exposure to violence, police protection)*

10. **Transport**
    - Safety *(Safety belts/child restraints, helmets, age curfews/graduated driver's license program, driving while intoxicated laws/enforcement, speed restriction/enforcement)*
    - Infrastructure *(Roads)*
    - Traffic Patterns *(Spatial location of jobs, traffic volume, carpooling)*
    - Vehicles *(Number and type of vehicles)*
    - Public Transportation *(Availability, density, efficiency, types of public transportation available, cohesiveness/integration of trips taken)*
    - Economic Issues *(Expenditures, spending on local roads vs. alternative transportation, percent of transit revenue from fares, insurance rates, commuter taxes)*
