# Supplementary material for: Why Parents Say No to Having Their Children Vaccinated against Measles: A Systematic Review of the Social Determinants of Parental Perceptions on MMR Vaccine Hesitancy
Source: Vaccines (Basel). 2023 May 2;11(5):926. doi: 10.3390/vaccines11050926 (PMC10224336; doi:10.3390/vaccines11050926)
Supplement: Supplementary file 1 [file vaccines-11-00926-s001.zip › Table S1 Systematic review search terms and databases..pdf]

Table S1: Systematic review search terms and databases.

Title/abstract/keyword search statements:	
	(vaccin* OR immuniz* OR immunis* OR inoculat*)
AND	(hesitan* OR refus* OR oppos* OR resist*)
AND	(parent* OR father* OR mother* OR guardian* OR caregiver* OR "care giver*" OR family OR families OR familial)
AND	(measles OR mmr OR rubeola)
MeSH terms mapped to keywords:	
	Vaccination Hesitancy, Vaccination Refusal, Vaccination, Immunization, Measles, Measles-Mumps-Rubella Vaccine, Parents, Fathers, Mothers, Caregivers
Databases searched:	
	Web of Science, Scopus, Medline/PubMed, Embase, CINAHL, PsycINFO, and ProQuest Dissertation & Theses Global
