# Supplementary material for: Why Parents Say No to Having Their Children Vaccinated against Measles: A Systematic Review of the Social Determinants of Parental Perceptions on MMR Vaccine Hesitancy
Source: Vaccines (Basel). 2023 May 2;11(5):926. doi: 10.3390/vaccines11050926 (PMC10224336; doi:10.3390/vaccines11050926)
Supplement: Supplementary file 1 [file vaccines-11-00926-s001.zip › Table S2 MMAT assessment of methodological quality.pdf]

Table S2: Assessment of the methodological quality of included studies using the Mixed Methods Appraisal Tool (MMAT) version 18 (Hong et al., 2018). The MMAT score represents the number of methodological quality criteria met for a particular study (highest quality score = 5, lowest quality score = 0). Of the included studies, 77% met all five quality criteria, 18% met four of the five criteria, and 5% met three of the five criteria. Explanations for the screening questions and methodological quality criteria are provided in the table footnote.*

Author, year [Reference]	Screening Question 1	Screening Question 2	Criterion 1	Criterion 2	Criterion 3	Criterion 4	Criterion 5	MMAT Score
**Qualitative Study Design**								
Bahta & Ashkir, 2015 [85]	Yes	Yes	Yes	Yes	Yes	Yes	Yes	5
Campeau, 2020 [86]	Yes	Yes	Yes	Yes	Yes	Yes	Yes	5
Danso-Odei, 2017 [87]	Yes	Yes	Yes	Yes	Yes	Yes	Yes	5
Duchsherer et al., 2020 [88]	Yes	Yes	Yes	Yes	Yes	Yes	Yes	5
Kadono, 2020 [89]	Yes	Yes	Yes	Yes	Yes	Yes	Yes	5
Kang et al., 2017 [90]	Yes	Yes	Yes	Yes	Yes	Yes	Yes	5
McDonald et al., 2019 [91]	Yes	Yes	Yes	Yes	Yes	Yes	Yes	5
Steiner, 2020 [92]	Yes	Yes	Yes	No	Yes	Yes	Yes	4
Ugale et al., 2021 [93]	Yes	Yes	Yes	Yes	Yes	Yes	Yes	5
Wharton-Michael & Wharton-Clark, 2020 [94]	Yes	Yes	Yes	Yes	Yes	Yes	Yes	5
**Quantitative Randomized Controlled Trials**								
Glanz et al., 2020 [50]	Yes	Yes	Yes	Yes	Yes	Yes	Yes	5
Moyer-Gusé et al., 2018 [64]	Yes	Yes	Yes	Yes	Yes	Can’t tell	Yes	4
Nyhan et al., 2014 [69]	Yes	Yes	Yes	Yes	Yes	Yes	Yes	5
**Quantitative Non-Randomized**								
Blackshire & Iyieg-buniwe, 2021 [33]	Yes	Yes	Yes	Yes	Yes	Yes	Yes	5
Fuchs, 2016 [47]	Yes	Yes	Yes	Yes	Yes	Yes	Yes	5
Lieu et al., 2015 [60]	Yes	Yes	Yes	Yes	Yes	Yes	Yes	5
Newcomer et al., 2021 [66]	Yes	Yes	Yes	Yes	Yes	Yes	Yes	5
**Quantitative Descriptive**								

Bardenheier et al., 2004 [31]	Yes	Yes	Yes	Yes	Yes	Yes	Yes	5
Baumgaertner et al., 2018 [32]	Yes	Yes	Yes	Yes	Yes	Yes	Yes	5
Blakeslee, 2014 [34]	Yes	Yes	Yes	Yes	Yes	Yes	Yes	5
Bonsu et al., 2021 [35]	Yes	Yes	Yes	Yes	Yes	No	Yes	4
Buckman et al., 2020 [36]	Yes	Yes	Yes	Yes	Yes	Yes	Yes	5
Cacciatore et al., 2016 [37]	Yes	Yes	Yes	Yes	Yes	Yes	Yes	5
Cataldi et al., 2016 [38]	Yes	Yes	Yes	Yes	Yes	Yes	Yes	5
Christianson et al., 2020 [39]	Yes	Yes	Yes	No	Yes	Yes	Yes	4
Cole et al., 2022 [40]	Yes	Yes	Yes	Yes	Yes	Yes	Yes	5
Dempsey et al., 2011 [41]	Yes	Yes	Yes	Yes	Yes	Yes	Yes	5
Doll et al., 2021 [42]	Yes	Yes	Yes	Yes	Yes	Yes	Yes	5
Flanagan-Klygis et al., 2005 [43]	Yes	Yes	Yes	Yes	Yes	Yes	Yes	5
Freed et al., 2010 [44]	Yes	Yes	Yes	Yes	Yes	Yes	Yes	5
Freeman et al., 2022 [45]	Yes	Yes	Yes	Yes	Yes	Yes	Yes	5
Frew et al., 2016 [46]	Yes	Yes	Yes	Yes	Yes	Yes	Yes	5
Gennaro et al., 2021 [48]	Yes	Yes	Yes	Yes	Yes	Yes	Yes	5
Gilkey et al., 2016 [49]	Yes	Yes	Yes	Yes	Yes	Yes	Yes	5
Gowda et al., 2013 [51]	Yes	Yes	Yes	No	Yes	Yes	Yes	4
Gowda et al., 2013 [52]	Yes	Yes	Yes	Yes	Yes	Yes	Yes	5
Gromis & Liu, 2020 [53]	Yes	Yes	Yes	Yes	Yes	Yes	Yes	5
Holroyd et al., 2021 [54]	Yes	Yes	Yes	No	Yes	No	Yes	3
Kempe et al., 2020 [55]	Yes	Yes	Yes	Yes	Yes	Yes	Yes	5
Kettunen et al., 2017 [56]	Yes	Yes	Yes	Yes	Yes	Can’t tell	Yes	4
Kim, 2016 [22]	Yes	Yes	Yes	Can’t tell	Yes	Yes	Yes	4
Langkamp et al., 2020 [57]	Yes	Yes	Yes	Yes	Yes	Yes	Yes	5
Lee et al., 2016 [58]	Yes	Yes	Yes	Yes	Yes	Can’t tell	Yes	4

Leonard, 2015 [59]	Yes	Yes	Yes	Yes	Can't tell	Yes	Yes	4
McNutt et al., 2016 [61]	Yes	Yes	Yes	Yes	Yes	Yes	Yes	5
Mergler et al., 2013 [62]	Yes	Yes	Yes	No	Yes	No	Yes	3
Mills & Nilsen, 2020 [63]	Yes	Yes	Yes	Yes	Yes	No	Yes	5
Navin et al., 2019 [65]	Yes	Yes	Yes	Yes	Yes	Yes	Yes	5
Nguyen et al., 2022 [67]	Yes	Yes	Yes	No	Yes	No	Yes	3
Nyathi et al., 2019 [68]	Yes	Yes	Yes	Yes	Yes	Yes	Yes	5
Opel et al., 2011 [70]	Yes	Yes	Yes	Yes	Yes	Yes	Yes	5
Opel et al., 2013 [71]	Yes	Yes	Yes	Yes	Yes	Can't tell	Yes	4
Philpot, 2015 [72]	Yes	Yes	Yes	Yes	Yes	Yes	Yes	5
Qian et al., 2020 [73]	Yes	Yes	Yes	Yes	Yes	Yes	Yes	5
Reuben et al., 2020 [74]	Yes	Yes	Yes	Yes	Yes	Yes	Yes	5
Rodriguez-Nava et al., 2020 [75]	Yes	Yes	Yes	Yes	Yes	Yes	Yes	5
Sahni et al., 2020 [76]	Yes	Yes	Yes	Yes	Yes	Yes	Yes	5
Salazar, 2021 [77]	Yes	Yes	Yes	Yes	Yes	No	Yes	4
Salmon et al., 2005 [78]	Yes	Yes	Yes	Yes	Yes	Can't tell	Yes	5
Salmon et al., 2009 [79]	Yes	Yes	Yes	Yes	Yes	No	Yes	4
Salmon et al., 2015 [12]	Yes	Yes	Yes	Yes	Yes	Can't tell	Yes	4
Smith et al., 2010 [80]	Yes	Yes	Yes	Yes	Yes	Yes	Yes	5
Smith et al., 2011 [81]	Yes	Yes	Yes	Yes	Yes	Yes	Yes	5
Williams et al., 2016 [82]	Yes	Yes	Yes	Yes	Yes	Yes	Yes	5
Wolf et al., 2016 [83]	Yes	Yes	Yes	Yes	Yes	Yes	Yes	5
Xu et al., 2021 [84]	Yes	Yes	No	No	Yes	Yes	Yes	3
**Mixed Methods**								
Downs et al., 2008 [95]	Yes	Yes	Yes	Yes	Yes	Yes	Yes	5
Estep & Greenberg, 2020 [96]	Yes	Yes	Yes	Yes	Yes	Yes	Yes	5

Gahr et al., 2014 [97]	Yes	Yes	Yes	Yes	Yes	Yes	Yes	5
Kennedy & Gust, 2008 [98]	Yes	Yes	Yes	Yes	Yes	Yes	Yes	5
Parker et al., 2006 [99]	Yes	Yes	Yes	Yes	Yes	Yes	Yes	5
Smith et al., 2009 [100]	Yes	Yes	Yes	Yes	Yes	Yes	Yes	5
Sugerman et al., 2010 [23]	Yes	Yes	Yes	Yes	Yes	Yes	Yes	5

[*]MMAT version 18 screening questions and methodological quality criteria by study design type (Hong et al., 2018).

Screening questions for all study types

1. Are there clear research questions? **2.** Do the collected data allow to address the research questions?

Qualitative methodological quality criteria

1. Is the qualitative approach appropriate to answer the research question? **2.** Are the qualitative data collection methods adequate to address the research question? **3.** Are the findings adequately derived from the data? **4.** Is the interpretation of results sufficiently substantiated by data? **5.** Is there coherence between qualitative data sources, collection, analysis and interpretation?

Quantitative randomized controlled trials methodological quality criteria

1. Is randomization appropriately performed? 2. Are the groups comparable at baseline? 3. Are there complete outcome data? 4. Are outcome assessors blinded to the intervention provided? 5 Did the participants adhere to the assigned intervention?

Quantitative non-randomized methodological quality criteria

1. Are the participants representative of the target population? **2.** Are measurements appropriate regarding both the outcome and intervention (or exposure)? **3.** Are there complete outcome data? **4.** Are the confounders accounted for in the design and analysis? **5.** During the study period, is the intervention administered (or exposure occurred) as intended?

Quantitative descriptive methodological quality criteria

1. Is the sampling strategy relevant to address the research question? **2.** Is the sample representative of the target population? **3.** Are the measurements appropriate? **4.** Is the risk of nonresponse bias low? **5.** Is the statistical analysis appropriate to answer the research question?

Mixed methods methodological quality criteria

1. Is there an adequate rationale for using a mixed methods design to address the research question? **2.** Are the different components of the study effectively integrated to answer the research question? **3.** Are the outputs of the integration of qualitative and quantitative components adequately interpreted? **4.** Are divergences and inconsistencies between quantitative and qualitative results adequately addressed? **5.** Do the different components of the study adhere to the quality criteria of each tradition of the methods involved?
