# Supplementary material for: Why Parents Say No to Having Their Children Vaccinated against Measles: A Systematic Review of the Social Determinants of Parental Perceptions on MMR Vaccine Hesitancy
Source: Vaccines (Basel). 2023 May 2;11(5):926. doi: 10.3390/vaccines11050926 (PMC10224336; doi:10.3390/vaccines11050926)
Supplement: Supplementary file 1 [file vaccines-11-00926-s001.zip › Table S3 Characteristics of studies included in the systematic review.pdf]

**Table S3.** Characteristics of studies included in the systematic review ($n$ = 115).

| Author, year [Reference] | Parental vaccine beliefs | Reasons for MMR vaccine hesitancy | Reasons for general vaccine hesitancy |
|---|---|---|---|
| **Quantitative Study Design** | | | |
| Bardenheier et al., 2004 [31] | Hesitancy, refusal, opposition to measles/MMR, Meningococcal, Hep B, and DTP/DTaP vaccines only | • Cost or access to vaccines<br>• General/other safety concerns<br>• Low perceived benefit/susceptibility<br>• Risk of autism<br>• Too many vaccines per visit/concerns with vaccine schedule<br>• Vaccine adverse reactions/hypersensitivity reactions | • Cost or access to vaccines<br>• General/other safety concerns<br>• Low perceived benefit/susceptibility<br>• Risk of autism<br>• Too many vaccines per visit/concerns with vaccine schedule<br>• Vaccine adverse reactions/hypersensitivity reactions |
| Baumgaertner et al., 2018 [32] | Hesitancy, refusal, opposition to measles/MMR, Influenza, and Pertussis only | • Concerns with vaccine components<br>• General/other safety concerns<br>• Mistrust of government and health officials<br>• Philosophical/moral objection<br>• Risk of autism<br>• Vaccine adverse reactions/hypersensitivity reactions | • General/other safety concerns<br>• Mistrust of government and health officials<br>• Philosophical/moral objection<br>• Vaccine adverse reactions/hypersensitivity reactions<br>• Vaccine efficacy concerns |
| Blackshire & Iyiegbuniwe, 2021 [33] | Hesitancy, refusal, opposition to all vaccines | • General/other safety concerns | • Cost or access to vaccines<br>• General/other safety concerns<br>• Vaccine adverse reactions/hypersensitivity reactions |
| Blakeslee, 2014 [34] | Hesitancy, refusal, opposition to all vaccines | • Risk of autism<br>• Too many vaccines per visit/concerns with vaccine schedule<br>• Vaccine adverse reactions/hypersensitivity reactions | • Concerns with vaccine components<br>• Cost or access to vaccines<br>• Low perceived benefit/susceptibility<br>• Overwhelms immune system<br>• Pain on injection site<br>• Preference for "natural immunity"<br>• Risk of autism<br>• Vaccine adverse reactions/hypersensitivity reactions<br>• Vaccine efficacy concerns |
| Bonsu et al., 2021 [35] | Hesitancy, refusal, opposition to all vaccines | • General/other safety concerns | • General/other safety concerns<br>• Risk of autism |
| Buckman et al., 2020 [36] | Hesitancy, refusal, opposition to all vaccines | • Risk of autism | • Philosophical/moral objection<br>• Risk of autism<br>• Vaccine adverse reactions/hypersensitivity reactions |
| Cacciatore et al., 2016 [37] | Hesitancy, refusal, opposition to all vaccines | • General/other safety concerns | • Too many vaccines per visit/concerns with vaccine schedule<br>• Vaccine adverse reactions/hypersensitivity reactions<br>• Vaccine efficacy concerns |
| Cataldi et al., 2016 [38] | Hesitancy, refusal, opposition to measles/MMR only | • Low perceived benefit/susceptibility<br>• Philosophical/moral objection<br>• Risk of autism<br>• Vaccine efficacy concerns | • Not specified |
| Christianson et al., | Hesitancy, refusal, | • General/other safety concerns | • Not specified |

| | | | |
|---|---|---|---|
| 2020 [39] | opposition to measles/MMR only | • Risk of autism<br>• Vaccine adverse reactions/hypersensitivity reactions | |
| Cole et al., 2022 [40] | Hesitancy, refusal, opposition to all vaccines | • Cost or access to vaccines<br>• General/other safety concerns<br>• Vaccine efficacy concerns | • Overwhelms immune system<br>• Too many vaccines per visit/concerns with vaccine schedule |
| Dempsey et al., 2011 [41] | Hesitancy, refusal, opposition to all vaccines | • General/other safety concerns | • General/other safety concerns<br>• Mistrust of government and health officials<br>• Low perceived benefit/susceptibility<br>• Too many vaccines per visit/concerns with vaccine schedule<br>• Vaccine adverse reactions/hypersensitivity reactions<br>• Vaccine efficacy concerns |
| Doll et al., 2021 [42] | Hesitancy, refusal, opposition to measles/MMR only | • General/other safety concerns | • Not specified |
| Flanagan-Klygis et al., 2005 [43] | Hesitancy, refusal, opposition to all vaccines | • Concerns with vaccine components<br>• Risk of autism | • General/other safety concerns<br>• Philosophical/moral objection<br>• Religious opposition<br>• Too many vaccines per visit/concerns with vaccine schedule<br>• Vaccine adverse reactions/hypersensitivity reactions |
| Freed et al., 2010 [44] | Hesitancy, refusal, opposition to all vaccines | • Cost or access to vaccines<br>• General/other safety concerns<br>• Low perceived benefit/susceptibility<br>• Philosophical/moral objection<br>• Preference for "natural immunity"<br>• Risk of autism<br>• Vaccine adverse reactions/hypersensitivity reactions<br>• Vaccine efficacy concerns | • Cost or access to vaccines<br>• General/other safety concerns<br>• Mistrust of government and health officials<br>• Low perceived benefit/susceptibility<br>• Philosophical/moral objection<br>• Preference for "natural immunity"<br>• Risk of autism<br>• Vaccine adverse reactions/hypersensitivity reactions<br>• Vaccine efficacy concerns |
| Freeman et al., 2022 [45] | Hesitancy, refusal, opposition to all vaccines | • General/other safety concerns | • Cost or access to vaccines<br>• Too many vaccines per visit/concerns with vaccine schedule |
| Frew et al., 2016 [46] | Hesitancy, refusal, opposition to all vaccines | • General/other safety concerns | • Vaccine adverse reactions/hypersensitivity reactions |
| Fuchs, 2016 [47] | Hesitancy, refusal, opposition to all vaccines | • General/other safety concerns | • General/other safety concerns<br>• Mistrust of government and health officials<br>• Low perceived benefit/susceptibility<br>• Risk of autism<br>• Vaccine adverse reactions/hypersensitivity reactions |
| Gennaro et al., 2021 [48] | Hesitancy, refusal, opposition to all vaccines | • Cost or access to vaccines | • Concerns with vaccine components<br>• Cost or access to vaccines<br>• General/other safety concerns<br>• Low perceived benefit/susceptibility |

| | | | |
|---|---|---|---|
| | | | • Pain on injection site<br>• Too many vaccines per visit/concerns with vaccine schedule<br>• Vaccine adverse reactions/hypersensitivity reactions |
| Gilkey et al., 2016 [49] | Hesitancy, refusal, opposition to all vaccines | • General/other safety concerns | • Mistrust of government and health officials<br>• Low perceived benefit/susceptibility<br>• Too many vaccines per visit/concerns with vaccine schedule<br>• Vaccine adverse reactions/hypersensitivity reactions<br>• Vaccine efficacy concerns |
| Glanz et al., 2020 [50] | Hesitancy, refusal, opposition to all vaccines | • General/other safety concerns | • Concerns with vaccine components<br>• General/other safety concerns<br>• Too many vaccines per visit/concerns with vaccine schedule<br>• Vaccine efficacy concerns |
| Gowda et al., 2013 [51] | Hesitancy, refusal, opposition to all vaccines | • General/other safety concerns<br>• Low perceived benefit/susceptibility<br>• Preference for "natural immunity"<br>• Risk of autism<br>• Too many vaccines per visit/concerns with vaccine schedule<br>• Vaccine adverse reactions/hypersensitivity reactions<br>• Vaccine efficacy concerns | • Low perceived benefit/susceptibility<br>• Overwhelms immune system<br>• Preference for "natural immunity"<br>• Risk of autism<br>• Too many vaccines per visit/concerns with vaccine schedule<br>• Vaccine adverse reactions/hypersensitivity reactions |
| Gowda et al., 2013 [52] | Hesitancy, refusal, opposition to measles/MMR only | • General/other safety concerns<br>• Low perceived benefit/susceptibility<br>• Vaccine adverse reactions/hypersensitivity reactions | • Not specified |
| Gromis & Liu, 2020 [53] | Hesitancy, refusal, opposition to all vaccines | • General/other safety concerns | • General/other safety concerns<br>• Philosophical/moral objection |
| Holroyd et al., 2021 [54] | Hesitancy, refusal, opposition to all vaccines | • Low perceived benefit/susceptibility | • Cost or access to vaccines<br>• Low perceived benefit/susceptibility<br>• Overwhelms immune system<br>• Philosophical/moral objection<br>• Preference for "natural immunity"<br>• Too many vaccines per visit/concerns with vaccine schedule<br>• Vaccine adverse reactions/hypersensitivity reactions |
| Kempe et al., 2020 [55] | Hesitancy, refusal, opposition to all vaccines | • General/other safety concerns | • Vaccine adverse reactions/hypersensitivity reactions |
| Kettunen et al., 2017 [56] | Hesitancy, refusal, opposition to all vaccines | • Philosophical/moral objection<br>• Too many vaccines per visit/concerns with vaccine schedule<br>• Vaccine adverse reactions/hypersensitivity reactions | • Cost or access to vaccines<br>• General/other safety concerns<br>• Overwhelms immune system<br>• Philosophical/moral objection<br>• Too many vaccines per visit/concerns with vaccine schedule<br>• Vaccine adverse reactions/hypersensitivity |

| | | | |
|---|---|---|---|
| | | | reactions |
| Kim, 2016 [22] | Hesitancy, refusal, opposition to all vaccines | • General/other safety concerns<br>• Vaccine adverse reactions/hypersensitivity reactions | • Overwhelms immune system<br>• Risk of autism<br>• Vaccine adverse reactions/hypersensitivity reactions |
| Langkamp et al., 2020 [57] | Hesitancy, refusal, opposition to all vaccines | • General/other safety concerns | • General/other safety concerns<br>• Too many vaccines per visit/concerns with vaccine schedule<br>• Vaccine adverse reactions/hypersensitivity reactions |
| Lee et al., 2016 [58] | Hesitancy, refusal, opposition to all vaccines | • General/other safety concerns | • General/other safety concerns<br>• Mistrust of government and health officials<br>• Philosophical/moral objection<br>• Too many vaccines per visit/concerns with vaccine schedule |
| Leonard, 2015 [59] | Hesitancy, refusal, opposition to all vaccines | • General/other safety concerns<br>• Mistrust of government and health officials<br>• Philosophical/moral objection<br>• Risk of autism<br>• Vaccine adverse reactions/hypersensitivity reactions | • General/other safety concerns<br>• Mistrust of government and health officials<br>• Low perceived benefit/susceptibility<br>• Overwhelms immune system<br>• Philosophical/moral objection<br>• Religious opposition<br>• Risk of autism<br>• Too many vaccines per visit/concerns with vaccine schedule<br>• Vaccine adverse reactions/hypersensitivity reactions |
| Lieu et al., 2015 [60] | Hesitancy, refusal, opposition to all vaccines | • General/other safety concerns | • Cost or access to vaccines<br>• Too many vaccines per visit/concerns with vaccine schedule |
| McNutt et al., 2016 [61] | Hesitancy, refusal, opposition to all vaccines | • General/other safety concerns | • General/other safety concerns<br>• Vaccine efficacy concerns |
| Mergler et al., 2013 [62] | Hesitancy, refusal, opposition to all vaccines | • General/other safety concerns<br>• Low perceived benefit/susceptibility<br>• Vaccine adverse reactions/hypersensitivity reactions<br>• Vaccine efficacy concerns | • General/other safety concerns<br>• Low perceived benefit/susceptibility<br>• Overwhelms immune system<br>• Too many vaccines per visit/concerns with vaccine schedule<br>• Vaccine adverse reactions/hypersensitivity reactions<br>• Vaccine efficacy concerns |
| Mills & Nilsen, 2020 [63] | Hesitancy, refusal, opposition to all vaccines | • Vaccine adverse reactions/hypersensitivity reactions | • Concerns with vaccine components<br>• Low perceived benefit/susceptibility<br>• Risk of autism<br>• Vaccine adverse reactions/hypersensitivity reactions |
| Moyer-Gusé et al., 2018 [64] | Hesitancy, refusal, opposition to measles/MMR only | • Risk of autism<br>• Too many vaccines per visit/concerns with vaccine schedule<br>• Vaccine adverse reactions/hypersensitivity reactions | • Not specified |

| | | | |
|---|---|---|---|
| Navin et al., 2019 [65] | Hesitancy, refusal, opposition to all vaccines | • General/other safety concerns<br>• Low perceived benefit/susceptibility<br>• Religious opposition | • Low perceived benefit/susceptibility<br>• Preference for "natural immunity"<br>• Religious opposition<br>• Too many vaccines per visit/concerns with vaccine schedule<br>• Vaccine adverse reactions/hypersensitivity reactions |
| Newcomer et al., 2021 [66] | Hesitancy, refusal, opposition to all vaccines | • General/other safety concerns | • Too many vaccines per visit/concerns with vaccine schedule |
| Nguyen et al., 2022 [67] | Hesitancy, refusal, opposition to all vaccines | • Cost or access to vaccines | • Cost or access to vaccines<br>• Mistrust of government and health officials<br>• Too many vaccines per visit/concerns with vaccine schedule<br>• Vaccine adverse reactions/hypersensitivity reactions<br>• Vaccine efficacy concerns |
| Nyathi et al., 2019 [68] | Hesitancy, refusal, opposition to all vaccines | • General/other safety concerns<br>• Low perceived benefit/susceptibility<br>• Mistrust of government and health officials | • Mistrust of government and health officials<br>• Vaccine adverse reactions/hypersensitivity reactions |
| Nyhan et al., 2014 [69] | Hesitancy, refusal, opposition to measles/MMR only | • Risk of autism<br>• Vaccine adverse reactions/hypersensitivity reactions | • Not specified |
| Opel et al., 2011 [70] | Hesitancy, refusal, opposition to all vaccines | • General/other safety concerns | • Cost or access to vaccines<br>• General/other safety concerns<br>• Mistrust of government and health officials<br>• Overwhelms immune system<br>• Pain on injection site<br>• Preference for "natural immunity"<br>• Risk of autism<br>• Too many vaccines per visit/concerns with vaccine schedule<br>• Vaccine adverse reactions/hypersensitivity reactions |
| Opel et al., 2013 [71] | Hesitancy, refusal, opposition to all vaccines | • General/other safety concerns | • General/other safety concerns<br>• Vaccine adverse reactions/hypersensitivity reactions |
| Philpot, 2015 [72] | Hesitancy, refusal, opposition to measles/MMR and DTaP only | • Risk of autism | • Cost or access to vaccines<br>• Risk of autism<br>• Vaccine adverse reactions/hypersensitivity reactions |
| Qian et al., 2020 [73] | Hesitancy, refusal, opposition to measles/MMR only | • Concerns with vaccine components<br>• Overwhelms immune system<br>• Risk of autism<br>• Too many vaccines per visit/concerns with vaccine schedule | • Not specified |
| Reuben et al., 2020 [74] | Hesitancy, refusal, opposition to all vaccines | • Risk of autism<br>• Vaccine adverse reactions/hypersensitivity reactions | • Mistrust of government and health officials |
| Rodriguez-Nava et | Hesitancy, refusal, | • Vaccine adverse reactions/hypersensitivity | • Philosophical/moral objection |

| | | | |
|---|---|---|---|
| al., 2020 [75] | opposition to all vaccines | reactions | • Religious opposition<br>• Vaccine adverse reactions/hypersensitivity reactions |
| Sahni et al., 2020 [76] | Hesitancy, refusal, opposition to all vaccines | • Concerns with vaccine components<br>• Risk of autism<br>• Vaccine adverse reactions/hypersensitivity reactions | • Concerns with vaccine components<br>• Low perceived benefit/susceptibility<br>• Mistrust of government and health officials<br>• Risk of autism<br>• Vaccine adverse reactions/hypersensitivity reactions |
| Salazar, 2021 [77] | Hesitancy, refusal, opposition to all vaccines | • General/other safety concerns | • Too many vaccines per visit/concerns with vaccine schedule<br>• Preference for "natural immunity"<br>• Vaccine adverse reactions/hypersensitivity reactions<br>• Vaccine efficacy concerns |
| Salmon et al., 2005 [78] | Hesitancy, refusal, opposition to all vaccines | • General/other safety concerns | • Concerns with vaccine components<br>• General/other safety concerns<br>• Mistrust of government and health officials<br>• Low perceived benefit/susceptibility<br>• Overwhelms immune system<br>• Pain on injection site<br>• Philosophical/moral objection<br>• Religious opposition<br>• Vaccine adverse reactions/hypersensitivity reactions<br>• Vaccine efficacy concerns |
| Salmon et al., 2009 [79] | Hesitancy, refusal, opposition to all vaccines | • Risk of autism<br>• Vaccine adverse reactions/hypersensitivity reactions | • Concerns with vaccine components<br>• General/other safety concerns<br>• Mistrust of government and health officials<br>• Low perceived benefit/susceptibility<br>• Overwhelms immune system<br>• Philosophical/moral objection<br>• Preference for "natural immunity"<br>• Religious opposition<br>• Risk of autism<br>• Too many vaccines per visit/concerns with vaccine schedule<br>• Vaccine adverse reactions/hypersensitivity reactions<br>• Vaccine efficacy concerns |
| Salmon et al., 2015 [12] | Hesitancy, refusal, opposition to all vaccines | • Concerns with vaccine components<br>• Cost or access to vaccines<br>• General/other safety concerns<br>• Mistrust of government and health officials<br>• Low perceived benefit/susceptibility<br>• Pain on injection site<br>• Too many vaccines per visit/concerns with vaccine schedule<br>• Vaccine adverse reactions/hypersensitivity reactions<br>• Vaccine efficacy concerns | • Cost or access to vaccines<br>• General/other safety concerns<br>• Mistrust of government and health officials<br>• Pain on injection site<br>• Vaccine adverse reactions/hypersensitivity reactions<br>• Vaccine efficacy concerns |

| | | | |
|---|---|---|---|
| Smith et al., 2010 [80] | Hesitancy, refusal, opposition to all vaccines | • General/other safety concerns | • Cost or access to vaccines<br>• General/other safety concerns<br>• Risk of autism<br>• Too many vaccines per visit/concerns with vaccine schedule<br>• Vaccine adverse reactions/hypersensitivity reactions<br>• Vaccine efficacy concerns |
| Smith et al., 2011 [81] | Hesitancy, refusal, opposition to all vaccines | • General/other safety concerns | • Cost or access to vaccines<br>• General/other safety concerns<br>• Mistrust of government and health officials<br>• Low perceived benefit/susceptibility<br>• Overwhelms immune system<br>• Too many vaccines per visit/concerns with vaccine schedule<br>• Vaccine adverse reactions/hypersensitivity reactions<br>• Vaccine efficacy concerns |
| Williams et al., 2016 [82] | Hesitancy, refusal, opposition to all vaccines | • General/other safety concerns | • Religious opposition |
| Wolf et al., 2016 [83] | Hesitancy, refusal, opposition to all vaccines | • Risk of autism | • Mistrust of government and health officials<br>• Pain on injection site<br>• Risk of autism<br>• Too many vaccines per visit/concerns with vaccine schedule<br>• Vaccine adverse reactions/hypersensitivity reactions |
| Xu et al., 2021 [84] | Hesitancy, refusal, opposition to all vaccines | • Mistrust of government and health officials | • Mistrust of government and health officials |
| **Qualitative Study Design** | | | |
| Bahta & Ashkir, 2015 [85] | Hesitancy, refusal, opposition to measles/MMR only | • Risk of autism | • Not specified |
| Campeau, 2020 [86] | Hesitancy, refusal, opposition to measles/MMR only | • Concerns with vaccine components<br>• Mistrust of government and health officials<br>• Risk of autism<br>• Vaccine adverse reactions/hypersensitivity reactions | • Not specified |
| Danso-Odei, 2017 [87] | Hesitancy, refusal, opposition to all vaccines | • General/other safety concerns<br>• Mistrust of government and health officials<br>• Low perceived benefit/susceptibility<br>• Religious opposition<br>• Risk of autism<br>• Vaccine adverse reactions/hypersensitivity reactions | • Cost or access to vaccines<br>• Low perceived benefit/susceptibility<br>• Not recommended by healthcare provider<br>• Vaccine adverse reactions/hypersensitivity reactions |
| Duchsherer et al., 2020 [88] | Hesitancy, refusal, opposition to all vaccines | • Risk of autism<br>• Mistrust of government and health officials | • Risk of autism<br>• Mistrust of government and health officials<br>• Vaccine adverse reactions/hypersensitivity reactions |

| | | | |
|---|---|---|---|
| Kadono, 2020 [89] | Hesitancy, refusal, opposition to all vaccines | • Risk of autism | • Risk of autism<br>• Mistrust of government and health officials<br>• Vaccine adverse reactions/hypersensitivity reactions |
| Kang et al., 2017 [90] | Hesitancy, refusal, opposition to all vaccines | • Risk of autism | • Mistrust of government and health officials<br>• Vaccine adverse reactions/hypersensitivity reactions |
| McDonald et al., 2019 [91] | Hesitancy, refusal, opposition to all vaccines | • Vaccine adverse reactions/hypersensitivity reactions | • Concerns with vaccine components<br>• Low perceived benefit/susceptibility<br>• Overwhelms immune system<br>• Risk of autism<br>• Too many vaccines per visit/concerns with vaccine schedule<br>• Vaccine adverse reactions/hypersensitivity reactions |
| Steiner, 2020 [92] | Hesitancy, refusal, opposition to all vaccines | • Risk of autism<br>• Religious opposition | • Mistrust of government and health officials<br>• Religious opposition<br>• Vaccine adverse reactions/hypersensitivity reactions |
| Ugale et al., 2021 [93] | Hesitancy, refusal, opposition to all vaccines | • Concerns with vaccine components<br>• Low perceived benefit/susceptibility<br>• Religious opposition<br>• Risk of autism<br>• Too many vaccines per visit/concerns with vaccine schedule | • Concerns with vaccine components<br>• General/other safety concerns<br>• Low perceived benefit/susceptibility<br>• Mistrust of government and health officials<br>• Overwhelms immune system<br>• Pain on injection site<br>• Religious opposition<br>• Risk of autism<br>• Too many vaccines per visit/concerns with vaccine schedule<br>• Vaccine adverse reactions/hypersensitivity reaction |
| Wharton-Michael & Wharton-Clark, 2020 [94] | Hesitancy, refusal, opposition to all vaccines | • Risk of autism | • Concerns with vaccine components<br>• Mistrust of government and health officials<br>• Risk of autism |
| **Mixed Methods Study Design** | | | |
| Downs et al., 2008 [95] | Hesitancy, refusal, opposition to all vaccines | • Pain on injection site<br>• Risk of autism<br>• Vaccine adverse reactions/hypersensitivity reactions | • Pain on injection site<br>• Vaccine adverse reactions/hypersensitivity reactions |
| Estep & Greenberg, 2020 [96] | Hesitancy, refusal, opposition to all vaccines | • General/other safety concerns | • Low perceived benefit/susceptibility<br>• Philosophical/moral objection<br>• Vaccine adverse reactions/hypersensitivity reactions |
| Gahr et al., 2014 [97] | Hesitancy, refusal, opposition to measles/MMR only | • Risk of autism | • Not specified |
| Kennedy & Gust, 2008 [98] | Hesitancy, refusal, opposition to all vaccines | • Religious opposition<br>• Vaccine adverse reactions/hypersensitivity reactions | • Cost or access to vaccines<br>• Mistrust of government and health officials<br>• Low perceived benefit/susceptibility<br>• Not recommended by healthcare provider |

| | | | |
|---|---|---|---|
| | | | • Philosophical/moral objection<br>• Preference for “natural immunity”<br>• Religious opposition<br>• Risk of autism<br>• Vaccine adverse reactions/hypersensitivity reactions |
| Parker et al., 2006 [99] | Hesitancy, refusal, opposition to measles/MMR only | • Vaccine adverse reactions/hypersensitivity reactions | • Not specified |
| Smith et al., 2009 [100] | Hesitancy, refusal, opposition to measles/MMR, influenza, and HPV only | • Concerns with vaccine components<br>• Risk of autism | • Concerns with vaccine components<br>• General/other safety concerns<br>• Mistrust of government and health officials<br>• Low perceived benefit/susceptibility<br>• Overwhelms immune system<br>• Philosophical/moral objection<br>• Religious opposition<br>• Risk of autism<br>• Vaccine adverse reactions/hypersensitivity reactions |
| Sugerman et al., 2010 [23] | Hesitancy, refusal, opposition to all vaccines | • Vaccine adverse reactions/hypersensitivity reactions | • General/other safety concerns<br>• Mistrust of government and health officials<br>• Low perceived benefit/susceptibility<br>• Preference for “natural immunity”<br>• Risk of autism<br>• Vaccine adverse reactions/hypersensitivity reactions |
| **Literature Reviews/Conceptual Papers** | | | |
| Ackerman & Serrano, 2015 [101] | Hesitancy, refusal, opposition to all vaccines | • Risk of autism | • General/other safety concerns<br>• Low perceived benefit/susceptibility<br>• Preference for “natural immunity”<br>• Risk of autism<br>• Vaccine adverse reactions/hypersensitivity reactions |
| Akojie, 2021 [102] | Hesitancy, refusal, opposition to all vaccines | • Risk of autism | • Low perceived benefit/susceptibility<br>• Risk of autism<br>• Vaccine adverse reactions/hypersensitivity reactions |
| Anderson & Bryson, 2020 [103] | Hesitancy, refusal, opposition to all vaccines | • Concerns with vaccine components<br>• Risk of autism | • General/other safety concerns<br>• Low perceived benefit/susceptibility<br>• Mistrust of government and health officials<br>• Overwhelms immune system<br>• Pain on injection site<br>• Preference for “natural immunity”<br>• Religious opposition<br>• Risk of autism<br>• Vaccine adverse reactions/hypersensitivity reactions |
| Blendell & Fehr, 2012 [104] | Hesitancy, refusal, opposition to all vaccines | • Low perceived benefit/susceptibility<br>• Risk of autism | • Concerns with vaccine components<br>• General/other safety concerns<br>• Low perceived benefit/susceptibility<br>• Not recommended by healthcare provider<br>• Pain on injection site |

| | | | |
|---|---|---|---|
| | | | • Risk of autism<br>• Too many vaccines per visit/concerns with vaccine schedule<br>• Vaccine adverse reactions/hypersensitivity reactions |
| Braun & O'Leary, 2020 [105] | Hesitancy, refusal, opposition to all vaccines | • General/other safety concerns | • Concerns with vaccine components<br>• General/other safety concerns<br>• Mistrust of government and health officials<br>• Overwhelms immune system<br>• Preference for "natural immunity"<br>• Risk of autism<br>• Too many vaccines per visit/concerns with vaccine schedule |
| Cawkwell & Oshinsky, 2016 [106] | Hesitancy, refusal, opposition to all vaccines | • Risk of autism | • Not recommended by healthcare provider |
| Chatterjee & O'Keefe, 2010 [107] | Hesitancy, refusal, opposition to all vaccines | • Risk of autism | • Concerns with vaccine components<br>• General/other safety concerns<br>• Overwhelms immune system<br>• Pain on injection site<br>• Philosophical/moral objection<br>• Religious opposition<br>• Risk of autism<br>• Vaccine adverse reactions/hypersensitivity reactions |
| Colgrove & Bayer, 2005 [108] | Hesitancy, refusal, opposition to all vaccines | • Risk of autism | • Concerns with vaccine components<br>• General/other safety concerns<br>• Mistrust of government and health officials<br>• Overwhelms immune system<br>• Philosophical/moral objection<br>• Risk of autism<br>• Too many vaccines per visit/concerns with vaccine schedule<br>• Vaccine adverse reactions/hypersensitivity reactions |
| de St. Maurice et al., 2018 [109] | Hesitancy, refusal, opposition to all vaccines | • General/other safety concerns<br>• Low perceived benefit/susceptibility<br>• Pain on injection site<br>• Risk of autism<br>• Vaccine adverse reactions/hypersensitivity reactions | • Concerns with vaccine components<br>• General/other safety concerns<br>• Mistrust of government and health officials<br>• Low perceived benefit/susceptibility<br>• Not recommended by healthcare provider<br>• Pain on injection site<br>• Preference for "natural immunity"<br>• Risk of autism<br>• Vaccine adverse reactions/hypersensitivity reactions |
| DeStefano & Shimabukuro, 2019 [110] | Hesitancy, refusal, opposition to measles/MMR only | • General/other safety concerns<br>• Risk of autism<br>• Vaccine adverse reactions/hypersensitivity reactions | • General/other safety concerns<br>• Philosophical/moral objection<br>• Religious opposition |
| Dubé et al., 2013 [111] | Hesitancy, refusal, opposition to all vaccines | • Risk of autism | • Cost or access to vaccines<br>• Mistrust of government and health officials<br>• Low perceived benefit/susceptibility<br>• Overwhelms immune system |

| | | | |
|---|---|---|---|
| | | | • Pain on injection site<br>• Philosophical/moral objection<br>• Preference for "natural immunity"<br>• Religious opposition<br>• Risk of autism<br>• Too many vaccines per visit/concerns with vaccine schedule<br>• Vaccine adverse reactions/hypersensitivity reactions |
| Dubé et al., 2014 [112] | Hesitancy, refusal, opposition to all vaccines | • Religious opposition<br>• Risk of autism | • Concerns with vaccine components<br>• Cost or access to vaccines<br>• General/other safety concerns<br>• Mistrust of government and health officials<br>• Pain on injection site<br>• Philosophical/moral objection<br>• Preference for "natural immunity"<br>• Religious opposition<br>• Too many vaccines per visit/concerns with vaccine schedule<br>• Vaccine adverse reactions/hypersensitivity reactions |
| Gilmour et al., 2011 [113] | Hesitancy, refusal, opposition to all vaccines | • Risk of autism | • Not specified |
| Glanz et al., 2015 [114] | Hesitancy, refusal, opposition to all vaccines | • General/other safety concerns | • Vaccine adverse reactions/hypersensitivity reactions |
| Glanz et al., 2016 [115] | Hesitancy, refusal, opposition to all vaccines | • Concerns with vaccine components<br>• General/other safety concerns<br>• Risk of autism<br>• Too many vaccines per visit/concerns with vaccine schedule | • Concerns with vaccine components<br>• General/other safety concerns<br>• Overwhelms immune system<br>• Pain on injection site<br>• Too many vaccines per visit/concerns with vaccine schedule<br>• Vaccine adverse reactions/hypersensitivity reactions |
| Gowda & Dempsey, 2013 [11] | Hesitancy, refusal, opposition to all vaccines | • General/other safety concerns<br>• Low perceived benefit/susceptibility<br>• Pain on injection site<br>• Preference for "natural immunity"<br>• Risk of autism<br>• Vaccine adverse reactions/hypersensitivity reactions<br>• Vaccine efficacy concerns | • General/other safety concerns<br>• Low perceived benefit/susceptibility<br>• Overwhelms immune system<br>• Pain on injection site<br>• Preference for "natural immunity"<br>• Too many vaccines per visit/concerns with vaccine schedule<br>• Vaccine adverse reactions/hypersensitivity reactions<br>• Vaccine efficacy concerns |
| Gupta et al., 2020 [116] | Hesitancy, refusal, opposition to measles/MMR only | • Mistrust of government and health officials<br>• Risk of autism<br>• Vaccine adverse reactions/hypersensitivity reactions | • Not specified |
| Healy & Pickering, 2011 [117] | Hesitancy, refusal, opposition to all vaccines | • Risk of autism | • Concerns with vaccine components<br>• General/other safety concerns<br>• Overwhelms immune system<br>• Pain on injection site<br>• Preference for "natural immunity" |

| | | | |
|---|---|---|---|
| | | | • Risk of autism<br>• Vaccine adverse reactions/hypersensitivity reactions |
| Hulsey & Bland, 2015 [118] | Hesitancy, refusal, opposition to all vaccines | • Risk of autism<br>• Vaccine adverse reactions/hypersensitivity reactions | • Cost or access to vaccines<br>• General/other safety concerns<br>• Overwhelms immune system<br>• Pain on injection site<br>• Risk of autism<br>• Vaccine adverse reactions/hypersensitivity reactions |
| Jacobson, 2012 [119] | Hesitancy, refusal, opposition to all vaccines | • Risk of autism | • Concerns with vaccine components<br>• Cost or access to vaccines<br>• General/other safety concerns<br>• Low perceived benefit/susceptibility<br>• Overwhelms immune system<br>• Pain on injection site<br>• Philosophical/moral objection<br>• Preference for "natural immunity"<br>• Religious opposition<br>• Risk of autism<br>• Too many vaccines per visit/concerns with vaccine schedule<br>• Vaccine adverse reactions/hypersensitivity reactions<br>• Vaccine efficacy concerns |
| Jacobson et al., 2015 [120] | Hesitancy, refusal, opposition to all vaccines | • General/other safety concerns<br>• Risk of autism | • Concerns with vaccine components<br>• Overwhelms immune system<br>• Pain on injection site<br>• Preference for "natural immunity"<br>• Vaccine adverse reactions/hypersensitivity reactions<br>• Vaccine efficacy concerns |
| Johnson, 2017 [121] | Hesitancy, refusal, opposition to all vaccines | • Risk of autism<br>• Vaccine adverse reactions/hypersensitivity reactions | • Concerns with vaccine components<br>• Cost or access to vaccines<br>• Mistrust of government and health officials<br>• Low perceived benefit/susceptibility<br>• Overwhelms immune system<br>• Pain on injection site<br>• Philosophical/moral objection<br>• Preference for "natural immunity"<br>• Religious opposition<br>• Risk of autism<br>• Too many vaccines per visit/concerns with vaccine schedule<br>• Vaccine adverse reactions/hypersensitivity reactions |
| Keeton & Chen, 2010 [122] | Hesitancy, refusal, opposition to all vaccines | • Concerns with vaccine components<br>• Risk of autism | • Cost or access to vaccines<br>• General/other safety concerns<br>• Mistrust of government and health officials<br>• Low perceived benefit/susceptibility<br>• Too many vaccines per visit/concerns with vaccine schedule<br>• Vaccine adverse reactions/hypersensitivity reactions<br>• Vaccine efficacy concerns |

| | | | |
|---|---|---|---|
| Kubin, 2019 [123] | Hesitancy, refusal, opposition to all vaccines | • Risk of autism | • General/other safety concerns<br>• Low perceived benefit/susceptibility<br>• Not recommended by healthcare provider<br>• Overwhelms immune system<br>• Pain on injection site<br>• Philosophical/moral objection<br>• Preference for "natural immunity"<br>• Religious opposition<br>• Risk of autism<br>• Too many vaccines per visit/concerns with vaccine schedule<br>• Vaccine adverse reactions/hypersensitivity reactions |
| Lantos et al., 2010 [124] | Hesitancy, refusal, opposition to all vaccines | • General/other safety concerns | • Mistrust of government and health officials<br>• Philosophical/moral objection<br>• Religious opposition<br>• Too many vaccines per visit/concerns with vaccine schedule<br>• Vaccine adverse reactions/hypersensitivity reactions<br>• Vaccine efficacy concerns |
| Loehr, 2020 [125] | Hesitancy, refusal, opposition to all vaccines | • Mistrust of government and health officials<br>• Pain on injection site<br>• Religious opposition<br>• Risk of autism | • Concerns with vaccine components<br>• Low perceived benefit/susceptibility<br>• Mistrust of government and health officials<br>• Overwhelms immune system<br>• Pain on injection site<br>• Preference for "natural immunity"<br>• Religious opposition<br>• Risk of autism<br>• Too many vaccines per visit/concerns with vaccine schedule<br>• Vaccine adverse reactions/hypersensitivity reactions |
| Luthy et al., 2016 [126] | Hesitancy, refusal, opposition to all vaccines | • Risk of autism | • Concerns with vaccine components<br>• General/other safety concerns<br>• Overwhelms immune system<br>• Preference for "natural immunity"<br>• Risk of autism<br>• Vaccine adverse reactions/hypersensitivity reactions<br>• Vaccine efficacy concerns |
| Marcus, 2020 [127] | Hesitancy, refusal, opposition to all vaccines | • Risk of autism | • Cost or access to vaccines<br>• General/other safety concerns<br>• Risk of autism<br>• Vaccine adverse reactions/hypersensitivity reactions |
| Olusanya et al., 2021 [128] | Hesitancy, refusal, opposition to all vaccines | • Risk of autism | • Cost or access to vaccines<br>• Too many vaccines per visit/concerns with vaccine schedule<br>• Vaccine adverse reactions/hypersensitivity reactions |
| Omer et al., 2009 [129] | Hesitancy, refusal, opposition to all | • General/other safety concerns<br>• Vaccine adverse reactions/hypersensitivity | • General/other safety concerns<br>• Low perceived benefit/susceptibility |

| | | | |
|---|---|---|---|
| | vaccines | reactions | • Not recommended by healthcare provider<br>• Risk of autism<br>• Too many vaccines per visit/concerns with vaccine schedule<br>• Vaccine adverse reactions/hypersensitivity reactions<br>• Vaccine efficacy concerns |
| Papachrisanthou & Davis, 2019 [130] | Hesitancy, refusal, opposition to all vaccines | • Risk of autism<br>• Vaccine adverse reactions/hypersensitivity reactions | • Concerns with vaccine components<br>• Low perceived benefit/susceptibility<br>• Overwhelms immune system<br>• Preference for "natural immunity"<br>• Risk of autism<br>• Too many vaccines per visit/concerns with vaccine schedule<br>• Vaccine adverse reactions/hypersensitivity reactions |
| Paquette, 2021 [131] | Hesitancy, refusal, opposition to all vaccines | • Concerns with vaccine components<br>• General/other safety concerns<br>• Overwhelms immune system<br>• Risk of autism | • Concerns with vaccine components<br>• General/other safety concerns<br>• Low perceived benefit/susceptibility<br>• Mistrust of government and health officials<br>• Overwhelms immune system<br>• Risk of autism<br>• Vaccine adverse reactions/hypersensitivity reactions |
| Pierik, 2017 [132] | Hesitancy, refusal, opposition to all vaccines | • Pain on injection site<br>• Religious opposition<br>• Risk of autism<br>• Vaccine adverse reactions/hypersensitivity reactions | • Concerns with vaccine components<br>• General/other safety concerns<br>• Pain on injection site<br>• Philosophical/moral objection<br>• Preference for "natural immunity"<br>• Religious opposition<br>• Vaccine adverse reactions/hypersensitivity reactions |
| Schmidt, 2019 [133] | Hesitancy, refusal, opposition to all vaccines | • Concerns with vaccine components<br>• Risk of autism<br>• Vaccine adverse reactions/hypersensitivity reactions | • Concerns with vaccine components<br>• General/other safety concerns<br>• Low perceived benefit/susceptibility<br>• Mistrust of government and health officials<br>• Philosophical/moral objection<br>• Preference for "natural immunity"<br>• Religious opposition<br>• Vaccine adverse reactions/hypersensitivity reactions |
| Siddiqui et al., 2013 [134] | Hesitancy, refusal, opposition to all vaccines | • Risk of autism | • Concerns with vaccine components<br>• General/other safety concerns<br>• Mistrust of government and health officials<br>• Low perceived benefit/susceptibility<br>• Overwhelms immune system<br>• Risk of autism<br>• Too many vaccines per visit/concerns with vaccine schedule<br>• Vaccine adverse reactions/hypersensitivity reactions |
| Smith, 2015 [135] | Hesitancy, refusal, opposition to all vaccines | • Risk of autism | • Concerns with vaccine components<br>• Mistrust of government and health officials<br>• Overwhelms immune system |

| | | | |
|---|---|---|---|
| | | | • Pain on injection site<br>• Preference for "natural immunity"<br>• Risk of autism<br>• Too many vaccines per visit/concerns with vaccine schedule<br>• Vaccine adverse reactions/hypersensitivity reactions |
| Smith, 2017 [136] | Hesitancy, refusal, opposition to all vaccines | • Risk of autism | • Concerns with vaccine components<br>• General/other safety concerns<br>• Mistrust of government and health officials<br>• Low perceived benefit/susceptibility<br>• Overwhelms immune system<br>• Preference for "natural immunity"<br>• Risk of autism<br>• Too many vaccines per visit/concerns with vaccine schedule<br>• Vaccine adverse reactions/hypersensitivity reactions |
| Tankwanchi et al., 2021 [137] | Hesitancy, refusal, opposition to all vaccines | • Risk of autism<br>• Vaccine adverse reactions/hypersensitivity reactions | • Vaccine adverse reactions/hypersensitivity reactions |
| Tokish & Solanto, 2020 [138] | Hesitancy, refusal, opposition to all vaccines | • Risk of autism | • Concerns with vaccine components<br>• General/other safety concerns<br>• Low perceived benefit/susceptibility<br>• Mistrust of government and health officials<br>• Overwhelms immune system<br>• Pain on injection site<br>• Preference for "natural immunity"<br>• Risk of autism<br>• Too many vaccines per visit/concerns with vaccine schedule<br>• Vaccine adverse reactions/hypersensitivity reactions<br>• Vaccine efficacy concerns |
| Williams, 2014 [139] | Hesitancy, refusal, opposition to all vaccines | • Concerns with vaccine components<br>• General/other safety concerns<br>• Risk of autism<br>• Vaccine adverse reactions/hypersensitivity reactions<br>• Vaccine efficacy concerns | • Concerns with vaccine components<br>• General/other safety concerns<br>• Mistrust of government and health officials<br>• Low perceived benefit/susceptibility<br>• Overwhelms immune system<br>• Philosophical/moral objection<br>• Preference for "natural immunity"<br>• Religious opposition<br>• Risk of autism<br>• Too many vaccines per visit/concerns with vaccine schedule<br>• Vaccine adverse reactions/hypersensitivity reactions<br>• Vaccine efficacy concerns |
| Wilson et al., 2014 [140] | Hesitancy, refusal, opposition to all vaccines | • Cost or access to vaccines<br>• Low perceived benefit/susceptibility<br>• Risk of autism<br>• Vaccine adverse reactions/hypersensitivity reactions<br>• Vaccine efficacy concerns | • Cost or access to vaccines<br>• General/other safety concerns<br>• Mistrust of government and health officials<br>• Low perceived benefit/susceptibility<br>• Vaccine adverse reactions/hypersensitivity reactions<br>• Vaccine efficacy concerns |

| | | | |
|---|---|---|---|
| Wood et al., 2003 [141] | Hesitancy, refusal, opposition to all vaccines | • General/other safety concerns | • Vaccine adverse reactions/hypersensitivity reactions |
