# Supplementary material for: Why Parents Say No to Having Their Children Vaccinated against Measles: A Systematic Review of the Social Determinants of Parental Perceptions on MMR Vaccine Hesitancy
Source: Vaccines (Basel). 2023 May 2;11(5):926. doi: 10.3390/vaccines11050926 (PMC10224336; doi:10.3390/vaccines11050926)
Supplement: Supplementary file 1 [file vaccines-11-00926-s001.zip › Table S4 Vaccine hesitancy and parental demographic variables.pdf]

Table S4. Vaccine hesitancy and parental demographic variables.

Demographic variable	Author, year [Reference] – Explanation
**Age** (Total articles = 15)	
Older parental age and higher vaccine hesitancy (7 articles)	
	• Langkamp et al., 2020 [57] – Parents of children with Down syndrome who refused all vaccines had Parent Attitudes about Childhood Vaccines (PACV) scores of ≥ 50, were White, older, middle class, and well-educated
	• Salazar, 2021 [77] – Vaccine-hesitant parents (north area Denver, Colorado) were all mothers, married, with two or more children (93%), completed at least some college, and were at least 30 years old
	• Salmon et al., 2005 [78] – Parents of children with NMEs were older than vaccinated children
	• Salmon et al., 2015 [12] – Older maternal age was associated with greater vaccine hesitancy
	• Siddiqui et al., 2013 [134] – Unvaccinated children were more likely to have mothers ≥ 30 years
	• Smith et al., 2010 [80] – Intentional vaccine delays were higher for children with mothers ≥ 30 years
	• Smith et al., 2011 [81] – Children whose parents delayed or refused vaccination were significantly more likely to have mothers ≥ 30 years of age
Younger parental age and higher vaccine hesitancy (4 articles)	
	• Cataldi et al., 2016 [38] – Younger mothers were less knowledgeable about recent measles outbreaks compared to older mothers
	• Fuchs, 2016 [47] – Younger maternal age was correlated with lower vaccination rates
	• Bonsu et al., 2021 [35] – Parents of children with Autism Spectrum Disorder (ASD) were 3.3 years younger and more likely to agree that "toxins in vaccines" caused developmental delays in their children; the odds of being vaccine-hesitant were 11.9 times greater among parents who agreed that toxins in vaccines caused their children's developmental delays
	• Reuben et al., 2020 [74] – Greater vaccine hesitancy was associated with younger parental age, lower parental education, lower trust in physicians, higher levels of religiosity, and higher disgust sensitivity among participants from the US, Canada, and UK
No relationship observed between parental age and vaccine hesitancy (4 articles)	
	• Lee et al., 2016 [58] – No differences in levels of distrust in government and healthcare providers were observed between parents of different age levels
	• Leonard, 2015 [59] – Parental age did not moderate the relationship between general trust in doctors/governments and attitudes toward MMR
	• Philpot, 2015 [72] – Maternal age was not significantly related to MMR vaccination completion
	• Salmon et al., 2009 [79] – Parents of children with NMEs did not differ in age from those of vaccinated children
**Race/ethnicity** (Total articles = 30)	
Non-Hispanic White race and higher vaccine hesitancy (15 articles)	
	• Blakeslee, 2014 [34] – White children had significantly higher odds of being unvaccinated against MMR than Hispanic children
	• Freed et al., 2010 [44] – White parents were more likely to refuse vaccines for their children than Hispanic parents
	• Keeton & Chen, 2010 [122] – Vaccine refusal was associated with maternal race/ethnicity, which was higher among White mothers
	• Kim, 2016 [22] – Personal belief exemptions were significantly higher among Whites than Hispanic or Asian Americans
	• Langkamp et al., 2020 [57] – Parents of children with Down syndrome who refused all vaccines had PACV scores of ≥ 50, were White, older, middle class, and well-educated
	• Lieu et al., 2015 [60] – White groups were more likely to be unvaccinated/undervaccinated
	• Omer et al., 2009 [129] – Unvaccinated children were more likely to be White

	• Opel et al., 2011 [70] – White parents were more likely to have under-vaccinated children than non-White parents<br>• Philpot, 2015 [72] – White parents were more likely to under-vaccinate their children than other groups<br>• Salmon et al., 2015 [12] – White race was associated with lower vaccination levels<br>• Siddiqui et al., 2013 [134] – White children were more likely to be unvaccinated than other groups<br>• Smith et al., 2010 [80] – Intentionally delaying vaccine administration was significantly higher among non-Hispanic White children than Hispanic and Black children<br>• Smith et al., 2011 [81] – Children, whose parents delayed or refused vaccination, were significantly more likely to be non-Hispanic White<br>• Sugerman et al., 2010 [23] – Parents of intentionally undervaccinated children tended to be White<br>• Wood et al., 2003 [141] – White parents were more likely to be hesitant to MMR
Non-White race and higher vaccine hesitancy (11 articles)	
	• Bahta & Ashkir, 2015 [85] – MMR vaccination rates were significantly lower for Somali American children than other groups<br>• Blakeslee, 2014 [34] – Black children had significantly higher odds of being unvaccinated against MMR than Hispanic children<br>• Campeau, 2020 [86] – Vaccine hesitancy among Somali refugees was primarily a trust issue due to unmet health needs including system issues such as food and housing insecurity and proximity to environmental toxins<br>• Cataldi et al., 2016 [38] – Racial and ethnically diverse mothers were less knowledgeable about recent measles outbreaks when compared to White mothers<br>• Christianson et al., 2020 [39] – Fear of measles following a measles outbreak was the major parental driver for under-vaccinated, close-knit Somalis in Minnesota to have their children vaccinated, but the perceived connection between MMR and autism remained a parental concern for 71% of the community<br>• Freed et al., 2010 [44] – Hispanic parents were more likely to believe that vaccines cause autism than White or Black parents; Hispanic parents were less likely to have refused vaccines for their children than Black parents<br>• Gahr et al., 2014 [97] – Somali American parents were less likely to have their children vaccinated<br>• Lieu et al., 2015 [60] – Black children were more likely to be unvaccinated/under-vaccinated than other non-White groups<br>• Nguyen et al., 2022 [67] – Among 25% vaccine-hesitant parents, the highest proportion of vaccine hesitancy, was among Blacks or Hispanics, high school educated or lesser, and among households below the poverty level<br>• Opel et al., 2011 [70] – Black parents were more likely to distrust their child's doctor than non-Black parents<br>• Tankwanchi et al., 2021 [137] – Among immigrant parents and families, particularly among Somalis and Poles, there was a high vaccine hesitancy for measles, flu, and HPV that was strongly associated with fear and misinformation on vaccine adverse effects, limited knowledge of diseases and vaccines, distrust in host country health system, language barriers, and religious objections
Other race/ethnicity findings (4 articles)	
	• Dempsey et al., 2011 [41] – Non-Black race was associated with higher odds of using an alternative vaccination schedule<br>• Salmon et al., 2005 [78] – Parents of children with NMEs and parents of vaccinated children were similar in terms of race<br>• Salmon et al., 2009 [79] – No difference in race observed between parents of children with NMEs and parents of vaccinated children<br>• Wolf et al., 2016 [83] – Foreign-born parents from Mexico or India were more likely to immunize their children than US-born parents
**Education** (Total articles = 39)<br>*Higher level of parental education and higher vaccine hesitancy* (17 articles)	
	• Fuchs, 2016 [47] – Some college or higher education levels were correlated with lower vaccination rates

- Gowda & Dempsey, 2013 [11] – College educated parents were more likely to refuse or have safety concerns with childhood vaccines
- Gupta et al., 2020 [116] – There was an inverse correlation between parents' levels of education and acceptance of vaccines, with the highly-educated and more informed on vaccine pitfalls being more likely to decline vaccination
- Keeton & Chen, 2010 [122] – Parents who refuse childhood vaccinations were more likely to come from well-educated communities
- Kim, 2016 [22] – Vaccination-refusing parents were more likely to have higher educational levels
- Langkamp et al., 2020 [57] – Parents of children with Down syndrome, who refused all vaccines, had PACV scores of ≥50, were White, older, middle-class, and well-educated
- Omer et al., 2009 [129] – Unvaccinated children were more likely to have mothers with a college education when compared to under-vaccinated children
- Opel et al., 2011 [70] – Parents with at least some college education were more likely to be concerned that childhood vaccines might not be safe
- Qian et al., 2020 [73] – Persistent vaccination delays in the US from 1998-2011, were driven by college-educated mothers whose exposure to negative vaccine information strengthened their biases more than the exposure to positive information (confirmatory bias)
- Salazar, 2021 [77] – Vaccine-hesitant parents (north area Denver, Colorado) were all mothers, married, with two or more children (93%), completed some college, and were at least 30 years old
- Salmon et al., 2005 [78] – Parents of children with NMEs were more likely to have higher than median level of education
- Salmon et al., 2015 [12] – Intentional vaccine delay was associated with higher maternal education level
- Siddiqui et al., 2013 [134] – Unvaccinated children were more likely to have college educated mothers
- Smith et al., 2010 [80] – Children whose parents intentionally delayed vaccination were significantly more likely to have a mother with at least some college education
- Smith et al., 2011 [81] – Children whose parents delayed or refused vaccination were significantly more likely to have a mother who was a college graduate
- Sugerman et al., 2010 [23] – Parents of intentionally under-vaccinated children tended to be college-educated
- Wolf et al., 2016 [83] – Foreign-born mothers from Russia or Ukraine with higher levels of education have children who were less likely to be immunized against measles

Higher level of parental education and lower vaccine hesitancy (1 article)

- Doll et al., 2021 [42] – The 2014 Disneyland measles outbreak was associated with a 3.9% increase in ≥ 1 dose of measles-containing vaccine (MV) coverage among children of college-educated mothers that may be partly attributable to the high awareness of the Disneyland outbreak and the subsequent change in parental decision to vaccinate among vaccine-hesitant parents

Lower level of parental education and higher vaccine hesitancy (15 articles)

- Bardenheier et al., 2004 [31] – Children of parents with lower levels of education were less likely to be up to date with measles vaccination
- Blakeslee, 2014 [34] – Children of mothers with less education had significantly higher odds of being unvaccinated
- Cacciatore et al., 2016 [37] – Lower maternal education levels were associated with less knowledge about measles outbreaks, higher levels of vaccine concerns, and delaying/forgoing recommended vaccinations
- Cataldi et al., 2016 [38] – Mothers with lower educations levels were less knowledgeable about recent measles outbreaks when compared to higher-educated mothers
- Doll et al., 2021 [42] – The 2014 Disneyland measles outbreak was associated with a 3.2% decrease in ≥ 1 dose of the measles-containing vaccine (MV) coverage among children of mothers with less than a high school degree across all races/ethnicities
- Downs et al., 2008 [95] – Parents with a focused understanding of vaccine mechanisms were slightly more likely to be college educated than those with a naïve understanding
- Freeman et al., 2022 [45] – Lower maternal education was associated with under-vaccination patterns suggestive of parental hesitancy

- Fuchs, 2016 [47] – Less than high school education levels were correlated with lower vaccination rates
- Gowda & Dempsey, 2013 [11] – Lower education levels were associated with greater distrust in the medical community and more concerns about vaccine safety
- Kempe et al., 2020 [55] – Lower parental education was significantly associated with hesitancy for routine childhood and influenza vaccines
- Nguyen et al., 2022 [67] – Among 25% vaccine-hesitant parents, the highest proportion of vaccine hesitancy, was among Blacks or Hispanics, high school educated or lesser, and among households below the poverty level
- Opel et al., 2011 [70] – Parents with a high school education or less were more likely to have children who were under-vaccinated for a greater mean percentage of days and were more likely to believe that getting sick with a vaccine-preventable disease (VPD) was better than to being vaccinated
- Philpot, 2015 [72] – Mothers with a college education were the most compliant in their child's MMR/DTaP vaccinations
- Reuben et al., 2020 [74] – Greater vaccine hesitancy was associated with higher levels of religiosity, younger parental age, lower parental education, lower trust in physicians, and higher disgust sensitivity among participants from the US, Canada, and UK
- Wolf et al., 2016 [83] – Foreign-born parents from Somalia with lower education levels were less likely to immunize their children against measles; foreign-born mothers from India with higher education levels were more likely to immunize their children against measles

No relationship observed between parental level of education and vaccine hesitancy (4 articles)

- Freed et al., 2010 [44] – No associations were observed between education level and parental perspectives on vaccines
- Lee et al., 2016 [58] – No differences in level of distrust in government and healthcare providers were observed between parents with high and low education levels
- Leonard, 2015 [59] – Parent education level did not moderate the relationship between general trust in doctors/governments and attitudes toward MMR
- Salmon et al., 2009 [79] – No differences in education level were observed between parents of children with NMEs and those with vaccinated children

Private and/or charter home-based schools and higher vaccine hesitancy (2 articles)

- Gromis & Liu, 2020 [53] – Private and charter schools had higher rates of personal belief exemptions (PBEs) from 2000-2015 compared to public schools; following the implementation of Senate Bill 277 (SB277), large increases in physician-granted permanent medical exemptions (PMEs) clustered in private and charter schools, which corresponded with the geographic locations of schools with previously high rates of PBEs in 2015
- Kadono, 2020 [89] – Parents enrolled their children in private or charter home-based education program to avoid vaccination requirements

Income/socioeconomic status (SES) (Total articles = 26)

Higher SES/income and higher vaccine hesitancy (12 articles)

- Dempsey et al., 2011 [41] – Non-mainstream beliefs about vaccination were more common among parents with higher incomes
- Estep & Greenberg, 2020 [96] – Vaccine refusal clustered in pockets of homogeneity, marked by high affluence, high exemption rates, false sense of lower disease vulnerability, which attracted parents who were more inclined to opt out of vaccinations
- Gowda & Dempsey, 2013 [11] – Parents in higher income brackets had greater concerns about vaccine safety
- Keeton & Chen, 2010 [122] – Parents who refuse childhood vaccinations were more likely to come from higher income communities
- Kim, 2016 [22] – Vaccination-refusing parents were more likely to have a high-income level
- McNutt et al., 2016 [61] – Affluence was associated with higher prevalence of personal belief exemptions
- Omer et al., 2009 [129] – Unvaccinated children were more likely to belong to high-income households when compared to under-vaccinated children
- Salmon et al., 2015 [12] – Intentional vaccine delay was associated with higher household income

- Siddiqui et al., 2013 [134] – Unvaccinated children were more likely to live in households with an annual income ≥ $75 000
- Smith et al., 2010 [80] – Children whose parents intentionally delayed vaccination were significantly more likely to live in a household with an annual income > $75,000
- Smith et al., 2011 [81] – Children whose parents delayed or refused vaccination were significantly more likely to live in a household with an annual income > 400% of the federal poverty level
- Sugerman et al., 2010 [23] – Higher public school NME rates were correlated significantly with higher median income

Lower SES/income and higher vaccine hesitancy (11 articles)

- Bardenheier et al., 2004 [31] – Children of parents with lower annual income were less likely to be up to date with measles vaccination
- Cataldi et al., 2016 [38] – Less wealthy mothers were less knowledgeable about recent measles outbreaks when compared to more affluent mothers
- Downs et al., 2008 [95] – Some parental failures to vaccinate were associated with low income
- Kempe et al., 2020 [55] – Lower household income at less than 400% of the federal poverty level was significantly associated with vaccine hesitancy for routine childhood and influenza vaccines
- Fuchs, 2016 [47] – Lower household income levels were correlated with lower vaccination rates
- Gennaro et al., 2021 [48] – The lack of stable housing makes it difficult for families experiencing homelessness to maintain an up-to-date vaccination status because of the difficulty in accessing primary care providers and in maintaining a continued relationship with health care provider
- Gowda & Dempsey, 2013 [11] – Parents in lower income brackets had greater vaccine opposition and concerns about vaccine safety
- Lee et al., 2016 [58] – Parents who distrust the government and healthcare providers had lower incomes than parents with high level of trust
- Nguyen et al., 2022 [67] – Among 25% vaccine-hesitant parents, the highest proportion of vaccine hesitancy was among Blacks or Hispanics, high school-educated or lesser, and among households below the poverty level
- Opel et al., 2011 [70] – Parents with a household income > $75,000 were more likely to be unconcerned with serious vaccine side effects and were less likely to agree that natural immunity is better than getting vaccinated
- Philpot, 2015 [72] – Mothers with a household income ≥ $75,000 were the most compliant in their child's MMR/DTaP vaccinations

No relationship observed between SES/income and vaccine hesitancy (3 articles)

- Opel et al., 2011 [70] – Parental income was not significantly associated with child's mean percentage of days under-vaccinated
- Salmon et al., 2005 [78] – Parents of children with NMEs and parents of vaccinated children had similar income levels
- Salmon et al., 2009 [79] – No differences in income levels were observed between parents of children with NMEs and those of vaccinated children

Health insurance (Total articles = 5)

- Cataldi et al., 2016 [38] – Mothers with private health insurance were more knowledgeable about recent measles outbreaks when compared to mothers without private insurance
- Freeman et al., 2022 [45] – Lack of insurance was associated with under-vaccination patterns suggestive of parental hesitancy
- Fuchs, 2016 [47] – Public insurance/no insurance was correlated with lower vaccination rates
- Smith et al., 2011 [81] – Parents with private health insurance were more likely to delay or refuse vaccines than those without private insurance
- Philpot, 2015 [72] – Mothers with health insurance were more likely to have their children vaccinated

Social influences/social network (Total articles = 2)

- Estep & Greenberg, 2020 [96] – Place-based ideological sorting as to socioeconomic status, political affiliations, or religious beliefs, created social enclaves of vaccine resistance that made it easier for parents to opt out because it was perceived to be safe and socially acceptable
- Gromis & Liu, 2020 [53] – Association between high PBEs and high PMEs areas may be due to the local spread of anti-vaccine beliefs and of the information on PME-granting physicians through parental social networks, which demonstrated continued parental resistance to school-entry vaccine requirements; parents residing in communities where the sociodemographic characteristics were associated with increased PBEs were more likely to seek PME; social norms on vaccination are influenced by the vaccination decisions of others within parents' social network

Policies/political affiliation (Total articles = 10)

- Baumgaertner et al., 2018 [32] – Political conservatives were less likely to have their children vaccinated against measles
- Buckman et al., 2020 [36] – There was a 1% increase in vaccine exemption rates in North Carolina among voters who were neither Republicans nor Democrats, suggesting that mistrust in social institutions extended beyond traditional political party platforms
- Gromis & Liu, 2020 [53] – Introduction of SB277, aimed at increasing vaccine compliance, may have had the unintended effect of increasing PMEs in private and charter schools
- Holroyd et al., 2021 [54] – Implementation of SB277 led to the elimination of nonmedical exemptions (NMEs) in California, but increased medical exemptions; 44% of parents of kindergarteners reported being vaccine-hesitant, harbored concerns and misconceptions about vaccines and public health authorities
- Kadono, 2020 [89] – Under-vaccinated children enrolled in private home school or independent study programs in California increased following SB277, particularly in five counties (Kern, San Diego, San Bernardino, Sonoma, and Stanislaus) to avoid vaccination requirements, which presented a gender impact as homeschooling was primarily carried out by mothers
- McDonald et al., 2019 [91] – Home-schooling mothers in California believed that SB277 was an infringement on parental rights; mothers' perception of vaccine risk affected vaccine decision-making; vaccine-hesitant and vaccine-accepting mothers expressed varying confidence in the safety and effectiveness of vaccines, were not confident that vaccination posed lower risks than VPD; while skeptical and refusing mothers generally believed that vaccinations were unsafe and ineffective, refused certain vaccines, believed that vaccination was riskier than VPDs, and belief of vaccine harm was a salient factor in vaccine decision-making
- Nyathi et al., 2019 [68] – SB 277 eliminated NMEs for childhood vaccines, including MMR, increased MMR coverage, and decreased non-medical exemptions (NMEs), but increased medical exemptions at the state and county levels; vaccine coverage was highest in counties with lower vaccine coverage before SB277
- Olusanya et al., 2021 [128] – Vaccine hesitancy and refusal mostly occurred due to state/local policies that allowed NMEs
- Paquette, 2021 [131] – A more contextualized approach was recommended vs. a broad restriction on vaccine exemptions, i.e., mandatory vaccination would be justified during an imminent public health threat and if an effective and safe vaccine was available; restrictions on vaccine exemptions would be justified if there were no less restrictive means to combat a public health threat and if restrictions were not disproportionately burdensome to any particular group
- Rodriguez-Nava et al., 2020 [75] – States in the US with higher NMEs, which was suggestive of vaccine hesitancy, had significantly lower MMR vaccine coverage due to misinformation on MMR vaccine safety despite absence of a disproportionate reporting on MMR-associated adverse drug reactions compared with all other vaccines in the past 30 years

Religion/religiosity (Total articles = 5)

- Marcus, 2020 [127] – Ultra-Orthodox Jewish communities in Brooklyn, New York, and in Rockland County, a large religious enclave in northern New York State, were the epicenters of the 2018-2019 measles outbreak in the US, had active ant-vaccine movement and widespread vaccine misinformation in their communities that contributed to under-vaccination
- Reuben et al., 2020 [74] – Greater vaccine hesitancy was associated with higher levels of religiosity, younger parental age, lower parental education, lower trust in physicians, and higher disgust sensitivity among participants from the US, Canada, and UK
- Schmidt, 2019 [133] – Ultra-orthodox Jewish communities Brooklyn, and Hudson Valley New York, US, who at times opposed vaccination, experienced more than 170 cases of measles in 2019

- Tankwanchi et al., 2021 [137] – Among immigrant parents and families, particularly among Somalis and Poles, there was a high vaccine hesitancy for measles, flu, and HPV; vaccine hesitancy was strongly associated with fear and misinformation of the adverse effects of vaccines, limited knowledge of diseases and vaccines, distrust of host country's health system, language barriers, and religious objections
- Ugale et al., 2021 [93] – Provider-reported reasons for parental refusal or delay were: (1) vaccine safety, (2) influence of information sources, decision-makers, timing, (3) low perceived risk for VPDs, (4) lack of trust, and (5) religious objections

Urban/rural areas (Total articles = 2)

- Newcomer et al., 2021 [66] – Majority of children received vaccines in clinics located outside of Montana's Metropolitan Statistical Areas (MSAs) and among these children, a significantly lower percentage completed the combined 7-vaccine series on time compared with children who received vaccines within MSAs; 18.7% of children had an under-vaccination pattern indicative of parental choice to delay or refuse vaccines in the form of restrictive shot-limiting, episodic shot-limiting, and selective vaccination
- Olusanya et al., 2021 [128] – Residing in certain metropolitan statistical areas (MSA) and non-MSA (mostly rural), and without health insurance were disproportionately less likely to be vaccinated; COVID-19 pandemic and the SDH of vaccine hesitancy exacerbated vaccination disparities

Chronic conditions (Total articles = 3)

- Bonsu et al., 2021 [35] – Parents of children with Autism Spectrum Disorder (ASD) were 3.3 years younger and more likely to agree that "toxins in vaccines" caused developmental delays in their children; the odds of being vaccine-hesitant were 11.9 times greater among parents who believed that toxins in vaccines caused their children's developmental delays
- Langkamp et al., 2020 [57] – Parents of children with Down syndrome who refused all vaccines had Parent Attitudes about Childhood Vaccines (PACV) scores of ≥ 50, were White, older, middle class, and well-educated
- Sahni et al., 2020 [76] – Vaccine hesitancy was highest among parents of children with ASD, who attributed ASD to toxins in vaccines compared to parents whose children had rheumatologic conditions and that of the general population; parents were less likely to be vaccine hesitant if they attributed their children's condition to chance or bad luck

Marital status (Total articles = 9)

Married parents and higher vaccine hesitancy (7 articles)

- Omer et al., 2009 [129] – Unvaccinated children were more likely to have married mothers compared to under-vaccinated children
- Philpot, 2015 [72] – Married mothers were less likely than unmarried mothers to have their children completely vaccinated with MMR/DTaP
- Salazar, 2021 [77] – Vaccine-hesitant parents (north area Denver, Colorado) were all mothers, married, with two or more children (93%), completed at least some college, and were at least 30 years old
- Siddiqui et al., 2013 [134] – Unvaccinated children were more likely to have married mothers
- Smith et al., 2010 [80] – Children whose parents intentionally delayed vaccination were significantly more likely to have married mothers
- Smith et al., 2011 [81] – Children whose parents delayed or refused vaccination were significantly more likely to have married mothers
- Wharton-Michael & Wharton-Clark, 2020 [94] – Parents who chose not to vaccinate their children were married stay-at-home mothers, whose education ranged from high school graduates to master's degrees, and used Google for online information to make vaccine decisions

Single parents and higher vaccine hesitancy (2 articles)

- Blakeslee, 2014 [34] – Children of currently unmarried mothers had significantly higher odds of being unvaccinated
- Fuchs, 2016 [47] – Unmarried marital status was correlated with lower child vaccination rates
